# Novel therapeutic approaches to target neurodegeneration

**DOI:** 10.1111/bph.16078

**Published:** 2023-04-26

**Authors:** Alerie G. de la Fuente, Silvia Pelucchi, Jerome Mertens, Monica Di Luca, Daniela Mauceri, Elena Marcello

**Affiliations:** ^1^ Instituto de Investigación Sanitaria y Biomédica de Alicante (ISABIAL) Alicante Spain; ^2^ Instituto de Neurociencias CSIC‐UMH Alicante Spain; ^3^ Wellcome‐Wolfson Institute for Experimental Medicine Queen's University Belfast Belfast UK; ^4^ Department of Pharmacological and Biomolecular Sciences University of Milan Milan Italy; ^5^ Institute of Molecular Biology Leopold‐Franzens‐Universität Innsbruck Innsbruck Austria; ^6^ Department of Neurosciences University of California San Diego La Jolla California USA; ^7^ Institute of Anatomy and Cell Biology Department of Molecular and Cellular Neuroscience, University of Marburg Marburg Germany; ^8^ Department of Neurobiology Interdisciplinary Centre for Neurosciences (IZN), Heidelberg University Heidelberg Germany

**Keywords:** autophagy, dendrite, gene expression, metabolism, neurodegenerative disorders, neuroinflammation, synapse

## Abstract

Ageing is the main risk factor common to most primary neurodegenerative disorders. Indeed, age‐related brain alterations have been long considered to predispose to neurodegeneration. Although protein misfolding and the accumulation of toxic protein aggregates have been considered as causative events in neurodegeneration, several other biological pathways affected by brain ageing also contribute to pathogenesis. Here, we discuss the evidence showing the involvement of the mechanisms controlling neuronal structure, gene expression, autophagy, cell metabolism and neuroinflammation in the onset and progression of neurodegenerative disorders. Furthermore, we review the therapeutic strategies currently under development or as future approaches designed to normalize these pathways, which may then increase brain resilience to cope with toxic protein species. In addition to therapies targeting the insoluble protein aggregates specifically associated with each neurodegenerative disorder, these novel pharmacological approaches may be part of combined therapies designed to rescue brain function.

**Abbreviations**
AD: Alzheimer's disease
ADAM10: a disintegrin and metalloproteinase 10
ALS: amyotrophic lateral sclerosis
AMPK: AMP‐activated protein kinase
APP: amyloid‐β precursor protein
AUTEN‐67: mTOR‐dependent modulator autophagy enhancer‐67
Aβ: amyloid‐β
CREB: cAMP response element‐binding protein
DDQ: methylphosphonate
FDA: Food and Drug Administration
FTD: frontotemporal dementia
HD: Huntington's disease
HDACs: histone deacetylases
MCU: mitochondrial calcium uniporter
Mdivi‐1: mitochondrial division inhibitor 1
Mfn1: mitofusin‐1
Mfn2: mitofusin‐2
MS: multiple sclerosis
mTOR: mammalian target of rapamycin
mTORC1: mTOR enzymatic complex 1
OMM: outer mitochondrial membrane
OPA1, optic: atrophy type 1
OPCs: oligodendrocyte progenitor cells
OPTN: optineurin
OXPHOS: oxidative phosphorylation
PD: Parkinson's disease
PINK1: PTEN‐induced protein kinase 1
PKM: pyruvate kinase M
RNF10: RING finger protein 10
ROS: reactive oxygen species
SASP: senescence‐associated secretory phenotype
SQSTM/p62: sequestosome‐1
TARDBP: (TAR)‐DNA‐binding protein

## 1 AGEING AND NEURODEGENERATIVE DISEASES

In the last century, advances in medical care and the creation of healthier environments have contributed to an increase in life expectancy (Niccoli & Partridge, 2012). Given that advanced age is the main risk factor for neurodegenerative diseases (Hou et al., 2019), any growth in the elderly population leads to a significant increase in the number of patients affected by age‐related primary neurodegenerative diseases, such as Alzheimer's disease (AD) (Hebert et al., 2010), Parkinson's disease (PD) (Poewe et al., 2017) or amyotrophic lateral sclerosis (ALS) (Talbott et al., 2016). Considering these current demographic changes, primary neurodegenerative diseases will have substantial socioeconomic implications for healthcare systems due to their high costs and marked effects on the quality of life of affected individuals and caregivers, posing a critical social emergency. Addressing these large burdens for society will require an intensified research approach and novel solutions (DiLuca & Olesen, 2014).

In the last 20 years, substantial advances have been made in our understanding of the pathogenesis of neurodegenerative disorders (Forman et al., 2004; Taylor et al., 2002). Much of this progress is the result of biochemical and histochemical characterization of proteins that accumulate within various inclusions in the diseased brain and genetic linkage studies identifying mutations in genes that cause neurodegenerative diseases. The identification of specific, disease‐segregating mutations in previously unknown genes directed the attention to proteins and pathways that are now considered crucial in the pathogenesis of neurodegenerative diseases. For instance, certain pathogenic mutations in the gene coding for the amyloid‐β precursor protein (APP) cause AD, in the α‐synuclein gene are related to PD, in huntingtin are related to Huntington's disease (HD) or in microtubule‐associated protein tau are associated with frontotemporal dementia (FTD) with parkinsonism (Bertram & Tanzi, 2005). The accumulation of species derived from these proteins in the brain of patients often represents a histological characteristic for each specific neurodegenerative disorder (Table 1).

**TABLE 1:** Main characteristics of the primary neurodegenerative disorders.

Neurodegenerative disease	Brain area affected	Misfolded proteins	Disease‐specific phenotype	Symptoms
Alzheimer's disease (AD)	Hippocampus Parietal and occipital lobes Entorhinal cortex Amygdala Locus coeruleus and raphe nucleus Basal forebrain	Tau Amyloid‐β	Synapse loss Neuronal atrophy Amyloid plaques Neurofibrillary tau tangles Vascular dysfunction	Memory decline Executive function decline Personality change Motor symptoms Communication deficits (aphasia)
Huntington's disease (HD)	Striatum Locus coeruleus Frontal cortex Putamen Caudate Basal ganglia	Huntingtin	Huntingtin polyglutamine (CAG) expansions Degeneration of spiny neurons Frontostriatal degeneration	Involuntary choreatic movements Cognitive and behavioural disturbances Hyperkinesia and later hypokinesia Dystonia Poor attention impulsivity and irritability Ataxia
Parkinson's disease (PD)	Substantia nigra Frontal cortex Brainstem	α‐Synuclein Tau	Loss of dopaminergic neurons Denervation of the nigrostriatal pathway Dystrophic neurites Presence of Lewy bodies	Tremor Rigidity Bradykinesia Postural instability Cognitive and communication
Frontotemporal dementia (FTD)	Frontal and temporal lobes Basal ganglia Brainstem	Transmembrane Protein 106B (TMEM106B) Tau Ubiquitin	Heterogeneous depending on the mutation type Neurocytoplasmic inclusions in superficial cortical layers	Unusual behaviours Emotional problems Trouble communicating (aphasia) Movement disorders
Amyotrophic lateral sclerosis (ALS)	Spinal cord and motor cortex	TDP‐43 Fus Optineurin Ubiquilin	Loss of motor neurons	Muscle weakness Motor deficits Progressive muscular atrophy

Despite the presence of these inherited cases, most neurodegenerative disorders develop sporadically in the absence of any known genetic aetiology. The onset of these sporadic forms is significantly influenced by risk factors (Bertram & Tanzi, 2005), ageing being the one with the highest impact on disease progression (Hou et al., 2019). Hence, age‐associated brain modifications are considered key contributors to the pathogenesis of neurodegenerative disorders (Daniele et al., 2018). However, the mechanistic interface(s) between brain ageing and neurodegeneration has not been fully elucidated. Since the 2000s, the ageing research field has grown considerably (Keshavarz et al., 2023). Understanding exactly how ageing increases the risk to develop neurodegenerative diseases can provide important clues for the development of new therapeutic strategies for the treatment of neurodegeneration.

**FIGURE 1:**
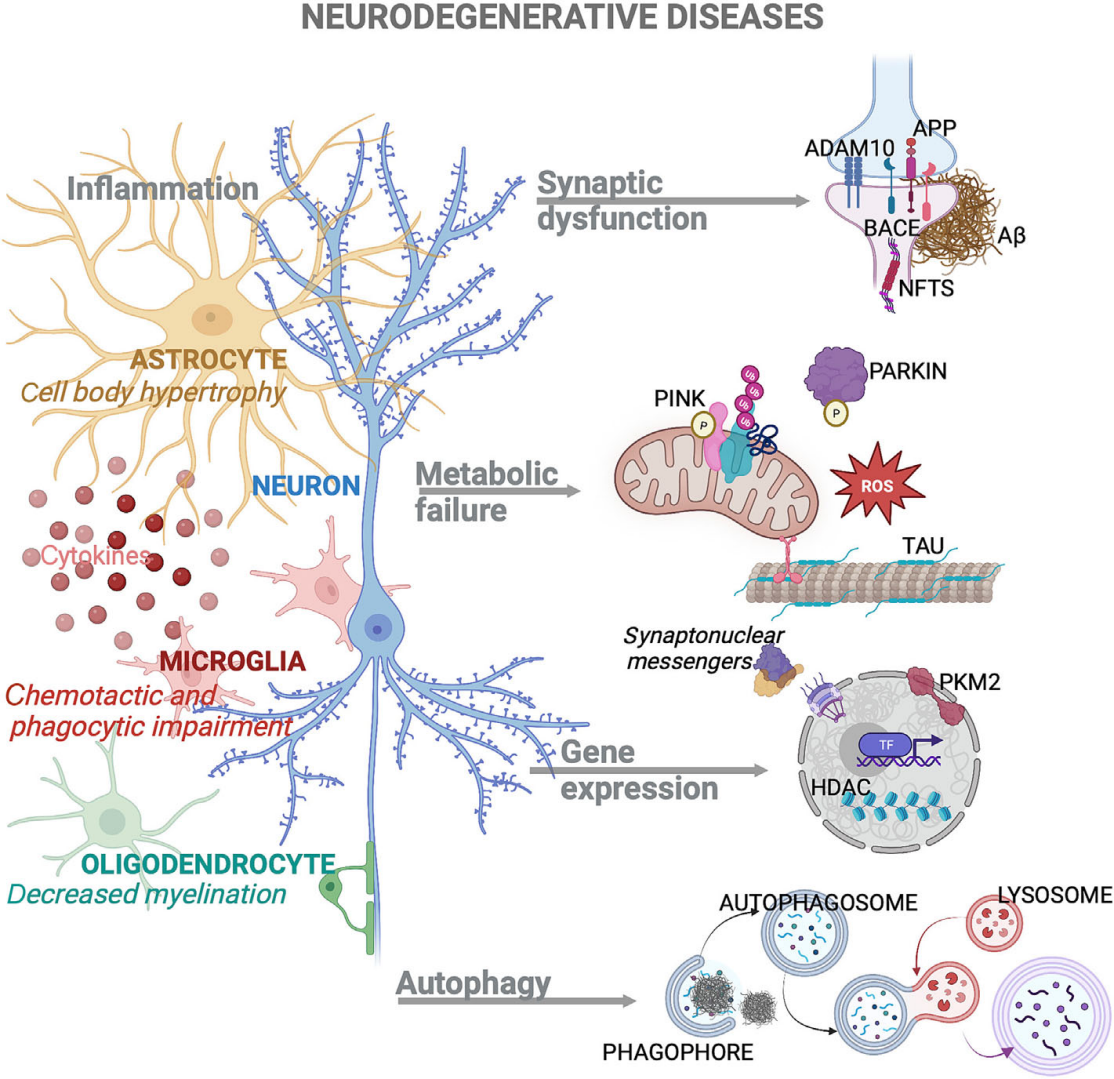
Summary of the biological pathways contributing to neurodegeneration.

Even though protein misfolding and the accumulation and formation of toxic protein species, due to inadequate folding, have been seen as causative events in neurodegenerative disorders, in this review, we carefully examine the role of other, different, biological pathways that are altered during ageing and implicated in the pathogenesis and progression of neurodegenerative disorders. We focus on the mechanisms controlling neuronal structure, gene expression, autophagy system, cell metabolism and, finally, neuroinflammation (Figure 1). Furthermore, we summarize the therapeutic approaches developed to restore these pathways that may increase the resilience of the brain to cope with toxic protein species, for each neurodegenerative disorder (Table 2).

**TABLE 2:** Summary of the therapeutic approaches developed to target the biological pathways contributing to the pathogenesis of primary neurodegenerative disorders.

Target mechanism	Drug	Mechanisms of action	Disease
Myelin	Clemastine fumarate	Binds to muscarinic receptor and activates OPC differentiation and myelination	*AD*, *ageing*
	Metformin	AMPK inhibitor, restores OPC differentiation and remyelination	*Ageing*
	LY294002	Modulator of PI3K–Akt–mTOR signalling modulator	*Ageing*
Synaptic dysfunction	Levetiracetam	Inhibits calcium release intraneuronal deposits Reverses synaptic dysfunction	**AD**
	Bryostatin 1	Activates PKC and regulates neurogenesis, axonal transport and synaptic plasticity	**AD**
	Masitinib	Tyrosine kinase inhibitor Mast cell inhibition, reduces secretion of toxic mediators for synapses	**AD** **ALS**
	Riluzole	Inhibits glutamate release	**ALS**
	Ceftriaxone	Up‐regulation of glutamate transporter	**ALS**
	Cell‐permeable peptides targeting ADAM10 trafficking	Blocks ADAM10 endocytosis, up‐regulating ADAM10 activity	*AD*
		Inhibition of SAP97‐mediated ADAM10 trafficking to synapse, decrease in ADAM10 activity	*HD*
Gene expression: Transcriptional regulation and epigenetic mechanisms	Memantine	Contrasts NF‐κB pro‐inflammatory activity	**AD**
	Tubastatin ACY‐1215 MPT0G211 5‐Aroylindoles	HDAC inhibitors: • Facilitate gene expression through chromatin remodelling • Restore memory function	*AD*
Autophagy	Rapamycin	Blocks mTORC1—Removing autophagy inhibition	*Ageing*, *AD*, **ALS**
	AUTEN‐67	mTOR‐dependent modulation of autophagy Increases autophagy	*AD*
	Metformin	Activates AMPK promoting autophagy and blocking mTORC1	*Ageing*, *AD*, *PD*, *HD*
	AUTOTAC	Removes protein aggregates using autophagy	*AD*
Metabolic failure	MitoQ CoQ10 MitoVItE MitoTEMPOL	Antioxidants: • Limit mitochondrial ROS production	*AD* *PD*
	Resveratrol	Antioxidant indirectly activating PGC‐1α	**PD**, **AD**, **ALS**, **HD**
	Mdivi‐1	Dpr1 inhibitor limiting mitochondrial division	*AD*
	Shikonin	PKM2 modulator, an apoptotic break	*AD*
	MCU inhibitors	Limit mitochondrial calcium uptake	*AD*, *PD*
CNS inflammation	Siponimod Fingolimod Natalizumab	Limit immune cell infiltration in the CNS	*ALS* *AD*, *PD* *HD*
	Minocycline	Anti‐inflammatory (controversial results between pre‐clinical models and clinical trials)	**AD**, **HD**, **ALS**, *PD*
	IL‐2/anti‐IL‐2 monoclonal antibody complexes	Treg expansion	*ALS*, *AD*
	Gene delivery to overexpress IL‐2 CNS	IL‐2 overexpression under GFAP promoter to expand Treg locally in CNS	*Ageing*
	Dasatinib Digoxin Quercetin AP20187	Senolytics: • Removal of senescent cells in the CNS (oligodendrocyte, astrocytes and microglia)	**AD** *Ageing*

*Note*: In the rightmost column, the indication of the disease for which the molecules were tested in pre‐clinical studies (in italics) and in clinical trials (in boldface type).

Abbreviations: AD, Alzheimer's disease; ADAM10, a disintegrin and metalloproteinase 10; ALS, amyotrophic lateral sclerosis; AMPK, AMP‐activated protein kinase; AUTEN‐67, mTOR‐dependent modulator autophagy enhancer‐67; AUTOTAC, AUTOphagy TArgeting Chimera; CNS, central nervous system; GFAP, glial fibrillary acidic protein; HD, Huntington's disease; HDAC, histone deacetylase; MCU, mitochondrial calcium uniporter; mTOR, mammalian target of rapamycin; mTORC1, mTOR enzymatic complex 1; OPC, oligodendrocyte progenitor cell; PD, Parkinson's disease; PGC‐1α, peroxisome proliferator‐activated receptor‐γ coactivator‐1α; PKM2, pyruvate kinase M2; ROS, reactive oxygen species; Treg, regulatory T cell.

## 2 THE STRUCTURAL DISINTEGRATION: HOW TO RESHAPE NEURONS AND SYNAPSES

The synapse is the biological locus responsible for the transmission of information between neurons. Neuron‐to‐neuron synapses are composed of a presynaptic and postsynaptic compartment, each with unique proteins and structures to facilitate excitatory and inhibitory neurotransmission.

The majority of synapses are found on dendrites, branch‐like extensions of a neuron that receive information from other neurons and carry it to the neuronal soma. The excitatory postsynaptic machinery is localized in dendritic spines, small protrusions from the dendrite shaft. Dendrites can support information processing at multiple spatial scales to integrate synaptic signals finally transformed into action potentials (Spruston et al., 2016). Neuronal synaptic structures are not static but highly dynamic. The ability of neurons to modify the efficacy of synaptic transmission and the synaptic structure in response to different stimuli is called synaptic plasticity (Citri & Malenka, 2008). Synaptic plasticity has been proposed to play a central role in the brain's capacity to incorporate transient experiences into persistent memory traces.

Astrocytes and microglia can transmit information and modulate synaptic activity (Schafer et al., 2013). Astrocyte processes encapsulate the synaptic cleft and ensure recycling of released neurotransmitters, release co‐factors important for physiological neuronal transmission and maintain tissue ion homeostasis. Furthermore, astrocytes are connected via gap junction‐coupled networks that synchronize neuronal activity within brain regions (Verkhratsky & Nedergaard, 2018). Microglia, the brain‐resident immune cells, on the other hand phagocytose inactive synapses and release co‐factors that are important for the induction and maintenance of synaptic plasticity (Rogers et al., 2011).

In addition to microglia and astrocytes, myelin, a passive insulating layer formed by oligodendrocytes that ensures fast saltatory conduction of action potentials, is also essential for neuroprotection providing physical axonal protection and trophic support. Despite being long considered a static component of the central nervous system (CNS), it has now been demonstrated that myelin has a plastic nature and myelin plasticity is required for motor learning, fear memory and conditioning (Xin & Chan, 2020).

### 2.1 The structure of brain cells during ageing and in neurodegenerative disorders: new perspectives to tackle synaptic and neuronal dysfunction

Several studies document changes in the molecular signature, morphology and function of brain cells with ageing. The principal, age‐related, alteration in neuronal structure involves a reduction in dendrite length and number, with a loss of various dendritic spines (Castelli et al., 2019). On the contrary, astrocytes undergo an increased expression of cytoskeletal proteins, cell body hypertrophy and a reduction in the number of long, slender processes with ageing (Rawji et al., 2023). Furthermore, aged microglial cells show a gradual decrease in function, most notably in chemotactic and phagocytic capacity. In particular, mouse studies have documented impairments in the ability of ageing microglia to phagocytose amyloid‐β (Aβ) fibrils and myelin debris (Rawji et al., 2016).

The age‐dependent altered function of glial cells reduces their ability to homeostatically nurture, protect and regenerate neurons, generating a more inflammatory microenvironment that consequently promotes neuron and synapse loss and therefore neurodegeneration. Most of these alterations are mild in healthy ageing but are exacerbated in a range of neurodegenerative diseases such as AD, PD, ALS and HD, where they contribute to or accelerate neurodegeneration, facilitate protein aggregate deposition and impair cognition and motor function by disrupting connective pathways (Ettle et al., 2016).

In addition, recent studies have revealed that myelin remodelling persists throughout the lifespan (Hill et al., 2018; Hughes et al., 2018). White matter and thus myelin volume declined after 13 months in mice and, in humans, after the age of 44–47 (Bartzokis et al., 2001) with myelin alterations that contribute to age‐linked functional decline being detected prior to neuronal loss. These alterations include widespread and diffuse myelin breakdown, degeneration and reduced myelin renewal (Safaiyan et al., 2016), decreased myelin stability associated with lipid peroxidation (Chia et al., 1983), formation of splits containing cytoplasm and myelin balloons or spheroids and accumulation of myelin debris, such as multi‐lamellar myelin fragments (Safaiyan et al., 2016).

Although we currently lack therapeutic approaches aimed at enhancing myelination in the clinic, recent advances and high‐throughput screening approaches have provided us with potential pro‐myelinating compounds. Clemastine fumarate, a muscarinic receptor antagonist that was identified in a pro‐remyelinating drug screening, has met clinically defined efficacy endpoints in a clinical trial in patients with multiple sclerosis (MS) (Green et al., 2017). Recent evidence has also shown that clemastine prevents age‐related myelin loss, neurodegeneration and cognitive decline in healthy ageing and in a mouse model of AD (Chen et al., 2021; Wang et al., 2020). Additionally, other drugs that have been shown to promote myelin repair in the context of MS, such as metformin or LY294002 (Neumann et al., 2019; Rivera et al., 2021), could also be beneficial in preventing myelin breakdown and degeneration with age or in other neurodegenerative diseases, but their efficacy in this context has yet to be investigated.

Neurodegenerative diseases are characterized by abnormalities in dendritic structure and synapse loss in different brain regions depending on the neurodegenerative disease (Südhof & Malenka, 2008). In HD, for example, synapse loss is mainly detected in the striatal brain region, which is linked with progressive movement dis‐coordination (Nithianantharajah & Hannan, 2013). There is growing evidence from ALS patients, FTD patients and animal models that suggest that synaptic dysfunction and alterations in dendritic branching begin very early in the disease, well before symptom onset and motor neuron death (Gelon et al., 2022).

In AD neurons, the dendritic tree undergoes a rapid decline with a decrease in the number of dendritic shafts, whereas the few remaining show fewer and shorter branches (Dickstein et al., 2007). Furthermore, synaptic loss in the hippocampus and neocortex is known to be an early process in AD and the main structural correlate with AD cognitive dysfunction.

Given that neuronal connections represent the hardware for appropriate cognitive abilities, therapeutic strategies aimed at preserving dendritic and synaptic connections could conceivably be useful in neurodegenerative diseases. The process of neurite repair to replenish the degenerated dendrites would involve regrowth and rewiring of the new connections within the network. The local molecular and cellular milieu in the CNS however opposes neurite growth and thus renders this approach particularly challenging (Liu & Jan, 2020). A better option could be to use strategies aimed at upholding neuronal dendritic integrity rather than promoting its regrowth. In this regard, promising findings have shown that specifically preserving dendritic architecture in mouse models of acute neurodegeneration (such as stroke) counteracted the loss of neurons, which is typically accelerated by disrupted connectivity and ultimately resulted in benefit at a functional level (Mauceri et al., 2020; Schlüter et al., 2020).

Synaptic loss is a common feature of neurodegenerative disorders and has been associated with the presence of the oligomeric forms of Aβ and α‐synuclein, which are considered toxic for synapses in AD and PD. The oligomers of α‐synuclein, but not its fibrils, contribute significantly to dopaminergic loss and neuronal cell death (Winner et al., 2011). Similarly, it has been shown that the Aβ oligomers cause synaptic loss and impair the mechanisms of synaptic plasticity (Walsh et al., 2002). Even though the molecular and cellular mechanisms underlying synaptic dysfunction in AD have not yet been fully elucidated, the 2022 drug pipeline for AD has shown that synaptic plasticity/neuroprotective agents, for which Phase 2 and Phase 3 clinical trials are currently ongoing, constitute 17% and 19% of all disease‐modifying therapies, respectively, indicating that significant efforts are being made in targeting these mechanisms (Cummings et al., 2022).

A systematic review analysed the efficacy profile of 12 published results of clinical trials investigating the safety and efficacy of disease‐modifying drugs targeting synaptic plasticity in dementia (Piscopo et al., 2022). This analysis showed, however, that only three molecules (levetiracetam, bryostatin 1 and masitinib) gave promising results.

The use of levetiracetam relies on the relationship existing between epilepsy and AD reported in the past decades. For instance, clinical trials have demonstrated a considerably higher incidence of seizures in AD patients than in matched control subjects (Amatniek et al., 2006) and aberrant excitatory activity has been observed in AD animal models (Palop et al., 2007). Levetiracetam is a second‐generation antiepileptic drug approved as an adjunct therapy for partial seizures. The mechanism of action seems to involve neuronal binding to the synaptic vesicle protein 2 A, inhibiting the release of calcium from intraneuronal deposits opposing the activity of negative modulators of GABA and glycine‐dependent currents and inhibiting excessive synchronized activity between neurons (Lyseng‐Williamson, 2011). An important role of this molecule also appears to be linked to synaptic plasticity. The administration of levetiracetam to AD mice was reported to reverse synaptic dysfunction (Sanchez et al., 2012). The initial clinical trials with levetiracetam showed limited results because of the high clinical heterogeneity of the enrolled cohort. On the other hand, a recent clinical trial found that levetiracetam was able to significantly improve cognitive status only in patients with cortical hyperexcitability. This suggests that preselection of AD patients presenting symptoms ranging from subclinical epileptiform activity to seizures and cortical network hyperexcitability could improve the capacity to identify therapeutic effects of levetiracetam (Palop et al., 2007).

Numerous reports imply a critical role of deficits in protein kinase C (PKC) signalling, in the pathogenesis of AD (Alkon et al., 2007). Intensive efforts have therefore focused on the development of strategies to foster PKC activity. In this context, bryostatin 1 has drawn attention because this macrocyclic lactone can activate PKC and thereby regulate neurogenesis, axonal transport and synaptic plasticity (Kim et al., 2012). The administration of bryostatin 1 in AD mice restored the number of dendritic spines in the hippocampal CA1 area (Hongpaisan et al., 2011). The results of Phase 1 and 2 trials are not clear but recently pooled analyses from two randomized clinical trials confirmed a significant cognitive restoration elicited by bryostatin 1, in the absence of memantine treatment (Thompson et al., 2022).

Masitinib, a tyrosine kinase inhibitor, is usually used in the treatment of mast cell tumours in animals. Besides playing a key role in innate immunity, mast cells have been involved in different neurological conditions and studies in animal models have shown that mast cell depletion in AD mice increases the immunoreactivity of synaptic markers (Jones et al., 2019). In line with these results, masitinib administration has a protective effect on synapses because of mast cell inhibition (Li et al., 2020) and reduction of the secretion of specific mediators potentially toxic to synapses (Li & Selkoe, 2020). Initial studies in patients reported that participants treated with masitinib had significant improvement in cognitive function (Piette et al., 2011). Masitinib is therefore being currently investigated in a multicentre Phase 3 trial for patients with mild‐to‐moderate AD (NCT01872598), and some beneficial effects in ALS have also been shown (Mora et al., 2020).

Excitotoxicity caused by an excessive activity of NMDA receptors promotes cell death and represents a potential mechanism of neurodegeneration. Therefore, pharmacological treatments of ALS patients are aimed at counteracting glutamate excitotoxicity. Riluzole, an inhibitor of glutamate release, was approved by the US Food and Drug Administration (FDA) in 1995. Despite being associated with a short survival benefit of 2–3 months (Miller et al., 2002), the subsequent adoption of riluzole as a treatment for ALS was perhaps reflective of the need for therapeutic options in the face of this devastatingly progressive disease (Dharmadasa et al., 2017). Remarkably, findings from several open‐label, non‐randomized, trials have suggested that the greatest benefit occurs at earlier disease stages (Zoing et al., 2006). It is therefore possible that riluzole's therapeutic benefit is likely to affect or activate different cellular pathways depending on the disease stage (Cheah et al., 2010). Another molecule investigated to counteract excitotoxicity is the antibiotic ceftriaxone, which causes the up‐regulation of glutamate transporter and decreases glutamate‐induced toxicity (Siciliano et al., 2010). A Phase 3 trial of ceftriaxone indicated an overall increase in survival of patients with ALS.

Overall, these strategies tackle synaptic failure and dysfunction as common molecular mechanisms across neurodegenerative disorders. Another challenge for drug discovery research will be to design tools and molecules specifically targeting the mechanisms underlying synaptic dysfunction or dendrite degeneration in each of the different neurodegenerative disorders. An example is the control of the trafficking of the disintegrin and metalloproteinase 10 (ADAM10).

This metalloprotease together with APP and the enzymes involved in the amyloid cascade are synaptic elements located at both presynaptic and postsynaptic sides and play a critical role in regulating synaptic function (Lundgren et al., 2020; Marcello et al., 2007). ADAM10 not only prevents Aβ generation but is also a shedding enzyme that cleaves adhesion molecules, such as N‐cadherin, and shapes spine morphology (Malinverno et al., 2010). Furthermore, ADAM10 synaptic localization and activity are finely tuned by synaptic plasticity phenomena (Marcello et al., 2013), and its synaptic abundance and activity towards APP are affected in the hippocampus of AD patients (Marcello et al., 2012). This is the result of an impairment of ADAM10 local forward trafficking that depends on the PKC‐regulated association with SAP97 and is also related to an increase in ADAM10 endocytosis (Marcello et al., 2013; Saraceno et al., 2014). Notably, alterations in ADAM10 have also been described in HD, with increased levels of the mature form of ADAM10 in the brain areas that predominantly degenerate in HD reported (Vezzoli et al., 2019) in mouse models of HD and in human HD brain samples (Vezzoli et al., 2019). Accumulation of active ADAM10 at the postsynaptic compartment leads to increased proteolysis of N‐cadherin, which is likely to promote synaptic instability in HD.

The detailed knowledge of the mechanisms involving ADAM10 in the pathogenesis of AD and HD is critical to design disease‐specific strategies to target synaptic failure in AD and HD. Indeed, the strategy to counteract synaptic failure in AD takes advantage of the administration of a cell‐permeable peptide that blocks ADAM10 endocytosis, up‐regulates its activity, restores synaptic function without affecting plaque deposition and rescues cognitive defects in AD mice (Musardo et al., 2022). On the other hand, the use of a peptide designed to inhibit SAP97‐mediated ADAM10 trafficking to the synapse (Marcello et al., 2007) normalizes ADAM10 activity and rescues cognitive deficits in HD mice (Vezzoli et al., 2019).

These data confirm that synaptic failure is a common trait of neurodegenerative disorders but highlight the importance of investigating the molecular mechanisms underlying synaptic dysfunction in each disorder to design disease‐tailored therapeutic strategies.

## 3 THE MAIN PLAYERS CONTROLLING GENE EXPRESSION DURING AGEING AND IN NEURODEGENERATIVE DISORDERS

A key aspect sustaining and enabling various forms of plasticity is the regulation of gene expression. Basal transcription ensures the mere survival of neurons as cells. To be functional computational units, neurons need to further adapt their transcriptional responses to physiological and pathological cues. To do so, neurons employ an array of regulatory elements, epigenetic mechanisms and transcription factors, all of which are modulated by diverse stimuli. One of the most prominent stimuli that neurons are specialized to adapt is synaptic activity and calcium signals. Among the many epigenetic mechanisms employed by neurons, the ones influencing chromatin accessibility—DNA methylation and post‐translational modifications of histone proteins—receive the most attention and are the best characterized. More recently, non‐coding RNAs have received increasing attention.

Given its key role in ensuring neural functions, it comes as no surprise that a long list of alterations in the transcriptional landscape or dysfunctions of molecular players has been associated with neurodegeneration. Mechanistically, the role of transcription factors or epigenetic regulators in the pathogenesis of neurodegenerative conditions is explained by their capability to affect genes directly involved in the pathology or in mediating co‐morbidities. Here, we highlight some of the better studied transcription factors and epigenetic regulators and their involvement in neurodegeneration.

cAMP response element‐binding protein (CREB) is a ubiquitous transcription factor playing multiple roles in the CNS. Several signalling cascades converge on CREB, which acts in cooperation with co‐factors such as CBP/p300. CREB is fundamental for memory and learning and also essential to neuronal survival and protection, as shown in vivo in mice (Jancic et al., 2009). Due to its broad expression, being downstream of different signalling events and driving expression of a myriad of critical neuronal genes, alterations of CREB activity and/or CREB expression have been indeed reported in neurodegenerative conditions such as AD or PD in cultured neurons, in vivo transgenic animal models and humans (Pugazhenthi et al., 2011; Xu et al., 2022).

Not all transcription factors are always localized to the nucleus; a considerable number of them regulate their expression by subcellular localization instead. Thus, failures in the nucleo‐cytoplasmic shuttling of transcription factors represent another possibility leading to transcriptional dysfunction in neurodegenerative conditions. The first transcription factor for which movement between the cytosol and the nucleus was described was NF‐κB. Activity of NF‐κB is prevented when sequestered in the cytosol. Alterations of NF‐κB localization have been observed in the proximity of plaques in post‐mortem samples from AD patients (Kaltschmidt et al., 1999) and also in dopaminergic neurons of PD patients (Hunot et al., 1997) and are tightly linked to inflammatory states.

Furthermore, synapse to nucleus shuttling of proteins with the capacity to modulate gene transcription is a prominent way in which inputs received at the synapse are transferred to the nucleus to implement long‐term changes. Synapses contain several nuclear localization signal‐containing cargo proteins and different components of the nuclear import machinery, like importin‐α and importin‐β, which have been shown to translocate to the nucleus in an activity‐dependent manner in primary neurons and hippocampal mouse slices (Thompson et al., 2004). In the last decade, a few synaptonuclear protein messengers (such as Abi‐1, AIDA‐1D, Jacob and RING finger protein 10 [RNF10]) have been identified and shown to play key roles in plasticity and synapse function (Fainzilber et al., 2011). Notably, the activation of NMDA receptors can regulate gene transcription in cultured neurons (Dieterich et al., 2008), thus affecting global protein synthesis and thereby memory formation. Such an effect of NMDA receptor activation requires the long‐distance trafficking of synaptonuclear proteins. The synaptonuclear messengers can associate with heterogeneous classes of receptors and specifically translate their activation in gene expression changes. For instance, the RNF10 operates as a mobile hub that docks GluN2A‐containing NMDA receptor‐derived signalosomes to nuclear target sites (Carrano et al., 2019; Dinamarca et al., 2016), whereas protein messengers, such as Jacob, can encode the synaptic and extrasynaptic origin of NMDA receptor signals following long‐distance transport and nuclear import in cultured primary neurons and in vivo in mice (Karpova et al., 2013). Remarkably, alterations in synaptonuclear messengers have been reported in neurodegenerative disorders such as AD (Marcello et al., 2018).

From the epigenetic perspective, reduced global methylation levels have been found in the blood of AD and PD patients in comparison to healthy controls, probably due to the decrease in expression of DNA methyltransferase 3α (Martínez‐Iglesias et al., 2020), a key mediator of this epigenetic mark in the context of signal‐regulated neuroepigenetics (Bayraktar & Kreutz, 2018). The scenario is, however, unclear as studies derived from post‐mortem samples of AD patients reported a reduction of global methylation levels in the cortex (Mastroeni et al., 2010) and hippocampus (Chouliaras et al., 2013); whereas in other studies, no changes (Lashley et al., 2015) or even an increase (Coppieters et al., 2014) in global DNA methylation have been observed. Studies focusing on DNA methylation changes at specific genomic regions, functional elements and individual gene loci have yielded a more comprehensive view in the context of AD and also revealed that epigenetic changes are complex, and often contradictory results are reported from different models (see Sanchez‐Mut & Gräff, 2015). Similarly, studies have indeed reported an association between PD disease progression and global methylation levels detected in the brain or blood or changes in the methylation pattern of specific genes (Henderson‐Smith et al., 2019).

Given the prominent role that histone deacetylases (HDACs) play in the modulation of several neuronal functions, it comes as no surprise that they have also been implicated in neurodegeneration. This heterogeneous group of proteins, classified based on activity, structure and co‐factors, is responsible for the removal of the acetyl group from histone as well as non‐histone proteins. An example of HDAC involvement in neuropathologies comes from HDAC2, HDAC6 or HDAC5, whose levels are elevated in brain areas of post‐mortem AD patients (Anderson et al., 2015; Gräff et al., 2012). Interestingly, an increased association between HDAC1 and CREB, possibly facilitating CREB pathological dephosphorylation, was observed in neuronal samples of PD patients (Xu et al., 2022). Expression levels of HDAC4, which has been implicated in many forms of pathologies of the nervous system in rodent models (Litke et al., 2022), were shown to be strongly associated with rapid progression of ALS in patients (Bruneteau et al., 2013). Alteration in the expression level of HDAC11 and HDAC2 was also reported in post‐mortem brain and spinal cord tissue of ALS patients (Janssen et al., 2010). Besides their action as transcriptional regulators, HDACs act also on cytosolic proteins of which the most prominent is tubulin. For example, HDAC6 acts on tubulin and has been associated with deficits in axonal transport in ALS patient‐derived motor neurons.

### 3.1 Therapeutic approaches to modify gene expression in neurodegenerative disorders

Targeting transcriptional regulators might be beneficial against neurodegenerative conditions as this would result in a widespread modulation of many affected downstream processes. This aspect however also represents a potential caveat as affecting the transcription of several genes might be detrimental as not all of them necessarily participate in the pathogenesis. Nevertheless, efforts have been made in exploring, designing and testing of therapeutic approaches aiming at modulating transcription‐related processes.

One of the most sought‐after targets in the treatment of neurodegenerative disorders is CREB due to the copious amount of evidence showing a reduction in its functionality or expression in many diseases. At present, however, there are no available drugs that specifically increase CREB‐dependent signalling. Multiple strategies could be followed, from acting on its upstream signalling regulators to using molecular biology or genetic approaches to restore its expression levels.

The complexity and multifactorial nature of many neurodegenerative disorders is likely to push the development of drugs towards targets different from the prototypical players associated with a certain disorder. An example of these types of targets is NF‐κB in the treatment of AD. Memantine, an FDA‐approved treatment for AD, seems to counter the pro‐inflammatory activity of NF‐κB. Additional anti‐inflammatory drugs are under pre‐clinical or clinical evaluation for their capacity to interfere with NF‐κB (see Sun et al., 2022).

Great efforts have been spent in the exploration of HDAC inhibitors as useful drugs in neurodegeneration. HDAC inhibitors may broadly facilitate gene expression via rendering the chromatin more permissive for transcription and thus enabling increased expression of potentially beneficial genes. One example of an AD‐relevant pathway, which could be positively regulated via HDAC modulation, is amyloid clearance. Indeed, pharmacological inhibition or genetic targeting of HDACs promoted amyloid clearance in humanized cultured astrocytes or different mouse AD models (Prasad & Rao, 2018; Su et al., 2021). Furthermore, in a mouse AD transgenic model, non‐selective HDAC inhibitors successfully restored memory function and neuronal structural aberrations (Ricobaraza et al., 2012). However, care should be taken to follow the path of non‐selective inhibition due to its potential side effects as shown in clinical studies (Prince et al., 2009). The design and development of specific HDAC inhibitors has been hindered by different problems including the fact that all HDACs share considerable structural similarities and are expressed across different organs and cell types. Nevertheless, with the advent of better technological opportunities, it is quite likely that inhibition of a specific HDAC may be a viable therapeutic avenue to pursue (Gupta et al., 2020). An encouraging example comes from the selective inhibition of HDAC6 by tubastatin A, ACY‐1215 (ricolinostat), MPT0G211 or 5‐aroylindoles, which have shown promising results in AD animal models (Fan et al., 2018; Lee et al., 2018; Onishi et al., 2021) and are currently under clinical trial evaluation for diseases other than neurodegeneration.

## 4 AUTOPHAGY: WHEN THE CLEARANCE SYSTEM FAILS AND DRIVES NEURODEGENERATION

Cellular homeostasis is the process that controls different cellular activities. It is a fundamental condition that allows the cell to maintain the physiological balance in terms of cellular identity, resilience and survival. Defects in protein homeostasis contribute to diminished degradation of intracellular proteins and organelles, which progressively accumulate in the cytoplasm. Such alterations therefore can be involved in the onset and progression of neurodegenerative disorders (Filippone et al., 2022). Degradation of cellular components within the cell can follow two different pathways: autophagy and the ubiquitin system, specific for protein degradation. Both processes require a concerted action of different proteins that are recruited and specifically recognize the damaged material, driving protein degradation as the last step to maintain cellular homeostasis. Under stress conditions such as ageing or disease, this highly complex process fails, resulting in a critical failure in cell physiology.

In mammalian cells, there are three types of autophagy: macroautophagy, microautophagy and chaperone‐mediated autophagy. Macroautophagy, simply known as autophagy, plays the major role in maintaining cellular homeostasis as it helps in the removal of bulky protein aggregates and bigger cytoplasmic bodies. It begins with the phagophore formation that entraps the misfolded proteins and, after different steps, finally fuses with lysosomes to generate the autophagolysosome (Wang & Hiesinger, 2012). Autophagy is tightly controlled by two kinases, the mammalian target of rapamycin (mTOR) and the AMP‐activated protein kinase (AMPK). Autophagy is promoted by AMPK, which is a key energy sensor and regulates cellular metabolism to maintain energy homeostasis (Liang et al., 2007). Conversely, autophagy is inhibited by mTOR, a central cell‐growth regulator that integrates growth factor and nutrient signals (Chang et al., 2009). Given its crucial role in cell homeostasis, a defect in autophagy is associated with neuronal loss and cognitive decline, both in physiological conditions such as ageing and in neurodegenerative diseases (Yamamoto & Simonsen, 2011). Interestingly, defects in the autophagy machinery have also been linked to axonal and dendritic degeneration in both in vitro and in vivo models and might therefore further promote the dysfunction of neural networks (Yang et al., 2013).

Several neurodegenerative diseases are characterized by defects in the degradation of misfolded proteins and thus abnormal protein aggregation. The impaired clearance of pathological proteins such as α‐synuclein, Aβ and tau, can be attributed to a failure of autophagy (Guo et al., 2018). For instance, Beclin‐1 is a protein involved in the regulation of autophagy and is reduced in AD patients (Lucin et al., 2013). Furthermore, the down‐regulation of Beclin‐1 in mice resulted in reduced neuronal autophagy and Aβ accumulation (Pickford et al., 2008). Moreover, the most common autosomal‐dominant form of PD and a familial variant that closely resembles sporadic PD is associated with mutations in the leucine‐rich repeat kinase 2 (LRKK2) (Orenstein et al., 2013). The altered function of mutated LRRK2 has been linked to defects in endosomal–lysosomal trafficking and chaperone‐mediated autophagy in cell lines (Gómez‐Suaga et al., 2012). In addition, in dopaminergic neurons, the lysosome number has been reported to be depleted in a mouse model of PD (Dehay et al., 2010).

Besides autophagy, cells take advantage of mitophagy, a specific process responsible for the selective degradation of dysfunctional mitochondria (Youle & Narendra, 2011). The PTEN‐induced protein kinase 1 (PINK1), localized on the external mitochondrial membrane, phosphorylates mitofusin‐2 (Mfn2) and ubiquitin, triggering the recruitment of the Parkin protein. This event activates several ubiquitin‐binding proteins such as optineurin (OPTN) and sequestosome‐1 (SQSTM/p62) that cause mitochondria to enter the mitophagy pathway. Defects in the mitophagy machinery are also a pathological marker of PD, and the accumulation of damaged mitochondria represents one of the main pathogenic alterations (Chu, 2010). Moreover, the PD‐associated familial autosomal recessive mutations in the genes for *PINK1* and *Parkin* have been discovered to have a key role in mitochondrial quality control (Malpartida et al., 2021). The phosphorylation of PINK1 at the outer mitochondrial membrane (OMM) leads to the recruitment of Parkin. Once active, Parkin allows the synthesis of ubiquitin chains on OMM proteins, leading to ubiquitin chain assembly. The mutations in the corresponding genes (*PINK1* and *PARK2*) are linked to autosomal recessive early‐onset PD and a pathological accumulation on the OMM that triggers an abnormal mitophagy (Zambon et al., 2019). In PD, α‐synuclein, the main component of the pathological Lewy bodies, has been found to bind mitochondrial components (Wang et al., 2019), inhibiting the import of proteins and leading to an impaired cellular respiration in primary neuronal cultures. In particular, α‐synuclein interacts with and disrupts mitochondrial proteins such as TOM20, the voltage‐dependent anion channels (VDAC) and F_1_Fo‐ATP synthase, thus impairing mitochondrial metabolism in dopamine neurons, derived from induced pluripotent stem cells (iPSC) (Zambon et al., 2019).

### 4.1 Targeting autophagy: how to promote protein and organelle clearance

In the last two decades, several studies have reported the neuroprotective activity of rapamycin (sirolimus), one of the most powerful pro‐autophagy agents in both cellular and animal models of neurodegenerative diseases and with some limitations in humans (Wang & Hiesinger, 2012). Rapamycin acts by blocking the kinase activity of mTOR enzymatic complex 1 (mTORC1), removing its autophagy suppressor activity that is observed under physiological conditions. The strategy of rapamycin treatment is to activate the autophagic flux which is negatively controlled by mTORC1. Interestingly, rapamycin has been proposed as an anti‐ageing drug in mice, because it was reported to increase the lifespan of the treated animal models (Selvarani et al., 2021). Rapamycin treatment has been also tested in animal models of AD where it reduced the accumulation of Aβ aggregates and prevented tau phosphorylation in the brain of AD transgenic mice, showing a global impact in cognition maintenance (Spilman et al., 2010). Regarding the use of rapamycin in humans, some encouraging results have been reported in the ALS field, where a recent, randomized, placebo‐controlled, Phase 2 clinical trial evaluated the efficacy of rapamycin in patients affected by ALS (Mandrioli et al., 2018).

Another approach to modulate the mTOR signalling level is possible using mTOR‐dependent modulator autophagy enhancer‐67 (AUTEN‐67), a small molecule identified as a potent candidate with anti‐ageing and neuroprotective effects, by significantly increasing autophagic flux in neurons and protecting them from undergoing stress‐induced cell death (Papp et al., 2016). Other agents that can indirectly trigger AMPK‐dependent mTOR inactivation are metformin and resveratrol, as shown in in vitro and in vivo models. Metformin activates AMPK, which in turn promotes autophagy, blocking mTORC1 activity through direct inhibitions of regulatory‐associated protein of mTOR. Moreover, the activation of AMPK directly promotes the activation of the phagophore‐forming enzymatic complex unc‐51‐like kinase (ULK)1/2, which is considered the initiator of the autophagic cascade (Thellung et al., 2019).

Furthermore, AUTOphagy TArgeting Chimera (AUTOTAC), a targeted protein degradation technology alternative to PROteolysis TArgeting Chimera (PROTAC), has emerged as one of the most promising approaches to remove specific disease‐associated proteins using the autophagy machinery of the cells (Ji et al., 2022). In the last few years, the PROTAC approach has been extensively used, with several PROTAC molecules currently in clinical trials in the cancer field (Mullard, 2021). A PROTAC molecule targeting tau protein has also been developed. It is a chimera construct made of a tau‐binding peptide, a linker, a VHL‐binding peptide and a cell‐penetrating peptide. Interestingly, this molecule leads to a significant degradation of tau and reduced neurotoxicity of Aβ in cell lines, highlighting the therapeutic potential of this approach (Chu et al., 2016).

## 5 METABOLIC FAILURE AND ENERGY CRISIS OF BRAIN CELLS

Among the different brain‐resident cell types, neurons are extremely energy demanding. Neurons rely almost exclusively on the mitochondrial oxidative phosphorylation (OXPHOS) system to fulfil their energy needs through ATP. The OXPHOS‐mediated mitochondrial functions are diverse, ranging from the cell‐intrinsic energy production to the regulation of intracellular calcium homeostasis, synaptic plasticity and neurotransmitter synthesis (Grimm & Eckert, 2017). On the other hand, this important energy production is accompanied by the formation of reactive oxygen species (ROS), which in excess are detrimental for cells.

ROS derive mainly from the process of the OXPHOS, which reduces O_2_ into H_2_O using the electron flux deriving from the respiratory chain and leading to the formation of superoxide anion radicals. Even if cell‐intrinsic antioxidant defence systems can buffer ROS, when this buffering system is overloaded and cell‐homeostasis altered, ROS become toxic. Given the fundamental role of mitochondria in neuronal energy supply, their dysfunction leads to an impairment of basal neuronal energy source, affecting several aspects of brain physiology. In addition to their metabolic activity, mitochondria play a key role in cellular calcium homeostasis. Cellular calcium concentration is strictly regulated as it sustains vital neuronal aspects such as secretion, motility, metabolic regulation, synaptic plasticity, proliferation, gene expression and apoptosis (Rizzuto et al., 2012). Mitochondrial calcium dysregulation therefore contributes to neurodegeneration as it is the major mechanism by which increased excitatory neurotransmission triggers mitochondrial depletion and retraction of dendritic structures (Verma et al., 2022).

The function of mitochondria is strictly related to their structure and dynamics. Thus, mitochondrial efficiency is measured through their capacity to undergo continuous fusion and fission cycles. Mitochondrial dynamism is therefore important for their morphology and function and relevant for neuronal viability and synaptic activity. The equilibrium between fission and fusion is key for adequate mitochondrial function and is compromised in different neurodegenerative disorders. The GTPase that controls mitochondrial fission, Drp1, is altered in AD, leading to an excessive fragmentation of mitochondria and thereby altering their function (Wang et al., 2009). This peculiar phenotype has been shown in neuronal cultures upon Aβ exposure as well as in several neurodegenerative disease animal models (Manczak et al., 2019; Wang et al., 2008). Defects in the dynamin‐related GTPase proteins mitofusin‐1 (Mfn1) and Mfn2 and atrophy type 1 (OPA1) protein affect the process of mitochondrial fusion and have been reported in various neurodegenerative disorders, including AD (Koshiba et al., 2004).

Alterations in mitochondrial trafficking, function and positioning in dendrites and synapses have been also observed in ALS and FTD (Sasaki & Iwata, 2007) and may contribute to early synaptic loss in disease (Gao et al., 2019). In FTD, several pathways controlling mitochondrial trafficking, dynamics and, consequently, activity are altered (Anoar et al., 2021). In the genetic FTD caused by *MAPT* mutations, a decrease in mitochondria–tau interactions in iPSC‐derived neurons has been observed. Tracy and collaborators reported that neurons expressing the V337M mutation in the tau protein were characterized by alterations in mitochondria bioenergetics affecting the efficiency in maintaining ATP levels under prolonged energetic stress (Tracy et al., 2022). In addition, the P301L mutation in tau, which is known to cause tau hyperphosphorylation, decreases mitochondrial respiration and ATP production, leading to a global mitochondrial and oxidative impairment (David et al., 2005). Mutations in transactive response (TAR)‐DNA‐binding protein (TARDBP), coding for the TDP‐43 protein, are also associated with FTD. Interestingly, TDP‐43 inclusions aggregate outside the nuclear compartment and can directly affect mitochondrial dynamics and trafficking, both at the axonal and dendritic compartment, leading to functional impairment. The overexpression or reduction of TDP‐43, in different animal model systems, leads to mitochondrial dysfunction (Xia et al., 2016).

In general, an altered cellular metabolism is considered a hallmark of ageing (Kim et al., 2018). During cellular senescence, which is distinct from, but associated with, biological ageing, mitochondria exhibit numerous changes in their structure, dynamics and function. In senescent cells, a decrease in mitochondrial membrane potential, an increase in proton leakage and a lowered oxidative capacity have been observed. The consequence of these modified processes resides in an altered aged metabolic homeostasis, with a significant increase in ROS generation, diminished antioxidant defence and a decrease in ATP production. All these phenomena have a significant effect on neurons that are particularly sensitive to stress and to the accumulation of a senescent profile, typical of ageing (Kim et al., 2018). The pool of healthy mitochondria also tends to decrease with ageing.

In addition to the role played by mitochondria in neurons themselves, neuronal energy demand is also sustained by glial cells that are extremely flexible and respond to environmental changes, providing neurons with their required energy (Traxler et al., 2021). Every time neurons need energy to perform the highly energy consuming neuronal synaptic burst, the so‐called astrocyte–neuron lactate shuttle responds to this energy demand by creating an energy bridge by which astrocyte‐produced lactate is received by neurons (Chuquet et al., 2010; Sá et al., 2017). The astrocytic–neuronal metabolic bridge is supported by the capacity of astrocytes to convert GABA and glutamate, removed from the synaptic cleft, into glutamine, which is used as a precursor for refill synaptic vesicles or for oxidative‐phosphorylation via the tricarboxylic acid (TCA) cycle (Bak et al., 2006; Traxler et al., 2021).

Neuronal metabolic pathways are comparatively inflexible and believed to be strictly regulated. However, metabolic changes including state shifts and alterations in individual metabolites can have marked effects on the neuronal epigenome. This enables cells to adapt to environmental changes and also poses a risk, as energetic challenges may lead to highly consequential epigenetic alterations (Traxler et al., 2021). Indeed, the cell fate specification and the consequent cell identity are established by a highly specific epigenetic control, which must be also plastic to allow adaptation to the environment. For instance, high glucose levels produce high acetylCoA:CoA ratio that regulates histone acetyltransferase activity and contributes to increased chromatin accessibility and gene activation (Lee et al., 2014). Both the early TCA cycle intermediate α‐ketoglutarate and oxygen are co‐substrates for demethylases, affecting DNA and histone methylation and thereby changing transcription scenarios (Traxler et al., 2021). Moreover, metabolic enzymes can translocate directly to the nucleus in a splicing‐ and signalling‐dependent manner and act directly on histones triggering changes in transcription. This is the case with pyruvate kinase M (PKM) that translocates to the nucleus where it phosphorylates histone 3 and leads to a de‐repression of cell‐cycle and glycolytic genes (Li et al., 2018). In induced neurons directly converted from fibroblasts derived from patients with AD, a cancer‐like metabolic switch from neuronal OXPHOS to aerobic glycolysis is associated with a higher level of the PKM2 nuclear isoform, compared with levels of the physiological PKM1 in these neurons. PKM2 prevalence is associated with metabolic and transcriptional changes in these AD‐derived neurons, contributing to AD‐related neuronal defects. Overall, all these new findings are suggestive of the presence of a metabolic reprogramming towards an aerobic glycolytic profile in AD, through a Warburg effect (Traxler et al., 2022).

The epigenetic modulation of metabolism, influenced by the combination of pathology and ageing, could be primary or secondary to mitochondrial impairment (Traxler et al., 2022). In general, in the neurodegenerative context, there is an accumulation of macromolecular damage and metabolic reprogramming that leads to the damage of organelles, including mitochondria, and eventually to tissue dysfunction (Kim et al., 2018).

### 5.1 Targeting mitochondria as a therapeutic strategy for treating neurodegeneration

To reduce and to buffer mitochondrial dysfunction, the most used indirect therapies rely on the use of antioxidants that mitigate mitochondrial ROS production. Some of those compounds include the lipophilic MitoQ, CoQ10, MitoVitE, MitoTEMPOL (Dumont et al., 2011; Johri, 2021; Shinn & Lagalwar, 2021; Zhelev et al., 2013) and resveratrol, which indirectly activates peroxisome proliferator‐activated receptor‐γ coactivator‐1α (PGC‐1α) and induces mitochondrial biogenesis (Yadav et al., 2022). There are also compounds that modify mitochondrial dynamics such as mitochondrial division inhibitor 1 (Mdivi‐1) (Dai et al., 2020) and methylphosphonate (DDQ) (Kuruva et al., 2017). Mdivi‐1, an inhibitor of Dpr1, showed activity against the excessive mitochondrial fragmentation induced by Aβ (Reddy et al., 2017). A novel approach recently proposed suggested combining the antioxidant effect with epigenetic modulation. Shikonin, an anti‐cancer PKM2 modulator, acts on the metabolic shift caused by neuronal PKM2 in AD, acting as an apoptotic brake on mature neurons (Traxler et al., 2022).

Given the pathogenic role for excitatory mitochondrial calcium dysregulation in mediating sub‐lethal dendritic atrophy observed in chronic neurodegenerative diseases, inhibiting calcium uptake has been reported to be neuroprotective. The major protein complex involved in mitochondrial calcium uptake is the mitochondrial calcium uniporter (MCU) (Baughman et al., 2011), and thereby, MCU inhibitors are neuroprotective in different genetic models of chronic neurodegenerative diseases (Verma et al., 2022).

## 6 NEUROINFLAMMATION

In recent years, it has become clear that despite having many different primary causes, all neurodegenerative diseases share a common constituent —neuroinflammation. Inflammation is the first line in host defence against pathogens and essential to the body's healing processes. However, chronic or prolonged inflammation, as observed in ageing and further exacerbated in neurological diseases, is detrimental for tissue homeostasis. Neuroinflammation can be triggered by CNS‐resident immune and glial cells (such as microglia, astrocytes and oligodendrocyte lineage cells), cells from the peripheral innate or adaptive immune system (T cells, B cells and macrophages), meningeal inflammation or autoantibodies directed to the CNS.

### 6.1 Neuroinflammation in ageing and neurodegeneration

There is growing evidence of the presence of both innate and adaptive immune cells in the healthy CNS, where they have key roles in maintaining homeostasis and immunosurveillance, being associated with neurogenesis (Ziv et al., 2006), learning and memory (Brynskikh et al., 2008) and synaptic pruning (Pasciuto et al., 2020), among other functions. This tightly regulated immune–CNS interaction is, however, distorted during ageing and even more abruptly in neurodegenerative disorders, leading to pathological neuroinflammation and subsequent neurodegeneration (Mayne et al., 2020). Even though present at low levels in the healthy young CNS (Pasciuto et al., 2020), an increase in adaptive immune cell infiltration, mainly CD8^+^ T cells and to a lesser extent CD4 ^+^ T cells, has been observed in the neurogenic niches, the white matter and the optic nerve, with ageing. Enhanced T‐cell infiltration alters CNS‐resident cell function increasing the expression of interferon‐responsive genes in CNS stem and glial cells (neural stem cells, microglia and oligodendrocytes) and contributes to age‐related myelin degeneration, impaired neurogenesis and axonal degeneration (Dulken et al., 2019; Groh et al., 2021; Kaya et al., 2022). Increased CNS infiltration of peripheral immune cells with ageing may result from blood–brain barrier alterations, increased permeability and a decreased CNS perfusion and lymphatic drainage (Blau et al., 2012).

Similar changes in T‐cell infiltration have also been observed in a range of neurodegenerative disorders not considered primary autoimmune disorders, such as AD, PD and ALS. T‐cell numbers, particularly of CD8^+^ T cells, are raised in the post‐mortem CNS tissue of AD, ALS and PD patients (Togo et al., 2002) and altered T‐cell levels or subsets in the cerebrospinal fluid and peripheral blood of AD, PD and ALS patients (Gate et al., 2020). The role of adaptive immune cell‐mediated inflammation in AD remains controversial. Even if T‐cell depletion has played a beneficial role, reversing cognitive decline, increasing Aβ clearance and promoting neuronal survival (Laurent et al., 2017), other studies have described detrimental roles for T cells in AD pathology (Marsh et al., 2016). In PD models, on the other hand, mice lacking mature lymphocytes show attenuated dopaminergic cell loss (Brochard et al., 2009), whereas in an ALS mouse model, infiltrates of CD8^+^ T cells in the CNS are associated with motor neuron loss (Coque et al., 2019). The role of T cell‐mediated neuroinflammation in neurodegenerative diseases therefore may be subset‐ and context‐specific, highlighting the complexity of the CNS–immune crosstalk and the role of neuroinflammation in neurodegeneration.

Prolonged CNS immune infiltration together with the enhanced production of pro‐inflammatory cytokines , such as IFN‐γ, TNF‐α, IL‐6 or IL‐1β, described in both ageing and neurodegenerative diseases also contribute to neurodegeneration by indirectly perpetuating inflammation through the priming of CNS glial cells. Single‐cell sequencing analysis of CNS‐resident cells, such as microglia, astrocytes and oligodendrocyte lineage cells in different neurodegenerative disease contexts and ageing, has unveiled disease‐specific phenotypes characterized by the expression of inflammatory and neurotoxic markers such as *Clec7a* , *C3* , *Lgals3* and *Trem2* in microglia (Olah et al., 2020), *Serpina3n*, *Lcn2*, *Ifitm3*, *Timp1* and *Chi3l1* in astrocytes (Hasel et al., 2021), and *Serpina3n*, *C4b* or *Klk6* in oligodendrocyte lineage cells (Kenigsbuch et al., 2022). Beyond this disease‐specific phenotype, an elevated expression of interferon‐responsive/stimulatory genes (e.g., *Irf1*, *Irf7*, *Irf8*, *Isg15* and *Ifit3*) has also been described across glial cells in ageing and neuroinflammation (Mathys et al., 2019). Additionally, neuroinflammation enhances the expression of antigen‐presenting genes (e.g., *Cd74* , *B2m*, *Cd9*, *H2‐K1* and *H2‐D1*) and immune cell chemoattractant cues such as *Icam‐1* , *Ccl2* , Cxcl12 or *Ccl3* by all the main glial cells, which in turn further activate CNS‐infiltrating T cells, contributing to a positive feedback loop that perpetuates neuroinflammation and thus neurodegeneration (Mathys et al., 2019).

In addition to immune cell infiltration and CNS‐resident cell‐driven pro‐inflammatory reactions, another source of neuroinflammation is linked to the accumulation of senescent cells with ageing and in CNS pathology. Mounting evidence has demonstrated the accumulation of senescence markers such as P16, P21, YH2A.X, lipofuscin, GATA4 and high‐mobility group box protein 1 in microglia, oligodendrocyte progenitor cells (OPCs), oligodendrocytes, astrocytes and neurons with ageing and in pathology such as AD (Bussian et al., 2018). Senescent cells accumulate in aged tissues and contribute to the pathogenesis of a range of neurodegenerative diseases, at least in part, through their pro‐inflammatory senescence‐associated secretory phenotype (SASP) (Guerrero et al., 2021), which can propagate senescence to neighbouring cells in a paracrine manner and contributes to immune cell recruitment to eliminate senescent cells (Acosta et al., 2013). Hence, neuroinflammation and senescent cells establish an additional positive feedback loop exacerbating ageing and disease pathogenesis. Chronic inflammation, as observed in ageing and neurodegenerative disorders, enhances the appearance of senescent cells, which, in turn, further contribute to neuroinflammation by the secretion of pro‐inflammatory molecules. It is therefore plausible that either by eliminating senescent cells or by modulating SASP, we can limit neuroinflammation and prevent neurodegeneration.

### 6.2 Therapeutic approaches to counteract neuroinflammation

Several drugs that limit immune cell infiltration in the CNS have been developed in the context of MS such as siponimod, fingolimod or natalizumab (Tintore et al., 2019), but whether these drugs are beneficial in other primary neurodegenerative diseases, such as AD or ALS, is still under debate. Natalizumab is a monoclonal antibody that blocks the extravasation of immune cells in the CNS and has been proven successful in mouse models of ALS such as SOD1^G93A^ and TDP43^A315T^ where it has diminished astrocyte and microglia priming, increasing motor neuron number and survival (Garofalo et al., 2022). Natalizumab has also shown beneficial effects in pre‐clinical models of AD such as APP/PS1 and 3xTg mice. In APP/PS1 mice, natalizumab reduced pro‐inflammatory cytokines in the spleen, CD4 immunoreactivity and general inflammation in the CNS (Manocha et al., 2018). Similarly, in 3xTg‐AD mice, natalizumab improved memory and reduced microgliosis, Aβ load and tau hyperphosphorylation (Pietronigro et al., 2019). Fingolimod, on the other hand, is a structural sphingosine analogue and a modulator of sphingosine 1‐phosphate (S1P) receptors, that blocks immune cell migration outside primary lymphoid organs and thus reduces T‐ and B‐cell number in the circulation. Fingolimod has shown to promote survival and improve the phenotype in SOD1^G93A^ mice and is well tolerated by patients with ALS, although its efficacy in disease progression is yet to be evaluated (Potenza et al., 2016). In AD, on the other hand, fingolimod ameliorated Aβ neurotoxicity in neuronal cultures (Joshi et al., 2017) while reducing Aβ and neuronal loss and astrocyte and microglial activation and improving memory and learning deficits in 5xFAD mice (Aytan et al., 2016). Furthermore, in PD mouse models such as the MPTP mouse model and a model performed by intracerebral injections of 6‐hydroxydopamine, fingolimod attenuated neuroinflammation, neuronal loss and motor deficits (Zhao et al., 2017). Moreover, low doses of fingolimod improved motor function and reduced brain atrophy, leading to the extended survival of R6/2 mice, a mouse model of HD (di Pardo et al., 2014). Thus, even though drugs that limit immune cell trafficking to the CNS appear to have a beneficial effect and help prevent neurodegeneration in different mouse models, further pre‐clinical investigations followed up by clinical trials are needed before clearly establishing their benefits for patients in other neurodegenerative diseases beyond MS.

An alternative approach to limit not only immune cell‐mediated inflammation but also neuroinflammation mediated by CNS‐resident cells involves the use of other less specific anti‐inflammatory drugs such as minocycline or non‐steroidal anti‐inflammatory compounds. Minocycline is a broad‐spectrum antibiotic with important anti‐inflammatory properties, and as such, it has been studied for several years in mouse models of neurodegeneration. Even if minocycline has been proven successful in limiting neuroinflammation and in some cases neurodegeneration in mouse models of AD (Choi et al., 2007), PD (Wu et al., 2002) and ALS (Kriz et al., 2002), its beneficial effect in subsequent clinical trials has been less robust, with no clear neurocognitive improvement observed in AD or HD and disease worsening detected in ALS trials (Cudkowicz, 2010; Gordon et al., 2007; Howard et al., 2020). Although most of the pre‐clinical data using minocycline reported positive results, its current negative outcomes or even the symptom worsening observed in some clinical trials, questions its effectiveness as a therapy for neurodegenerative diseases (Romero‐Miguel et al., 2021). Other anti‐inflammatory therapies considered for neurodegeneration include non‐steroidal anti‐inflammatory drugs (NSAIDs). However, the potential beneficial effects observed in some animal models were not reproduced in clinical trials, and thus, their use as potential therapy for neurodegeneration dropped (Sastre & Gentleman, 2010).

Considering the negative impact of sustained inflammation, mammals have developed their own endogenous anti‐inflammatory brake, mediated by regulatory T cells (Tregs), a subset of immune cells with high immune suppressive capacity. Tregs are known to be either depleted or functionally impaired in several neurodegenerative disorders (Liston et al., 2022). As a result, systemic Treg expansion or Treg adoptive transfer has been considered as a potential therapeutic approach to tackle neuroinflammation and prevent neurodegeneration. Treg expansion through peripheral IL‐2/IL‐2 monoclonal antibody complexes or adoptive transfer upon ex vivo activation has rendered positive results in mouse models of ALS and AD such as SOD1^G93A^ mice (Sheean et al., 2018), 5xFAD‐Rag2Ko mice (Faridar et al., 2022), 3xTg‐AD mice (Baek et al., 2016) and APP/PS1 mice (Dansokho et al., 2016). Tregs protect motor neurons, suppress astrocytic and microglial immunoreactivity, reduce amyloid burden and restore cognitive dysfunction. Moreover, an inverse correlation was observed between Treg numbers and disease progression upon Treg expansion in ALS patients, suggesting a neuroprotective effect also in humans (Beers et al., 2017). Despite the ample evidence of a beneficial role for Tregs in neurodegenerative diseases, systemic Treg expansion has not been widely considered for clinical trials as it can lead to systemic immune suppression in patients of advanced age and already vulnerable to infections, limiting its therapeutic use. Recent investigations have developed a gene delivery approach to locally expand Treg in the CNS by overexpressing IL‐2 in astrocytes and thus avoiding systemic immune suppression. This adenoviral‐based gene delivery approach has rendered positive results in mouse models of MS, stroke and traumatic brain injury (Yshii et al., 2022). Even though still to be tested in primary neurodegenerative disorders such as ALS, PD or AD, this approach opens novel therapeutic avenues to harness Treg immunosuppressive capacity to limit CNS neuroinflammation and neurodegeneration.

Lastly, we review the potential use of senolytics to limit neuroinflammation and prevent neurodegeneration. The fact that mice genetically engineered to remove p16^INK4a+^ senescent cells show a decrease in age‐related pathologies in several tissues, together with an extended lifespan and health span (Baker et al., 2016), stimulated interest in the development of senolytics such as dasatinib, digoxin or quercetin, as potential therapeutic approaches for neurodegeneration (Guerrero et al., 2021). Senescent OPCs have been found around Aβ plaques in post‐mortem tissue of patients with mild cognitive impairment (MCI) or AD and in APP/PS1 mice. The elimination of senescent OPCs by the administration of the senolytic cocktail formed by dasatinib and quercetin decreased microglial activation, Aβ load and the concentration of inflammatory cytokines IL‐6, IL‐1β and TNF‐α and improved cognitive performance (Zhang et al., 2019). Additionally, elimination of senescent astrocyte and microglia via the administration of the senolytic AP20187 in MAPT^P301S^PS19 tau pathology mouse model prevented gliosis, hyperphosphorylation of tau and neurodegeneration and preserved cognitive function (Bussian et al., 2018). Similarly, removal of senescent microglia via AP20187 or dasatinib and quercetin administration prevented age‐related cognitive decline and neuroinflammation (Ogrodnik et al., 2021). Thus, senolytics prevent neurodegeneration in ageing and pre‐clinical models of AD, supporting their use in clinical trials with older adults suffering from MCI or early‐stage AD (NCT04685590, SToMP‐AD; NCT04785300, ALSENLITE) (Guerrero et al., 2021). Their beneficial effect in other neurodegenerative diseases such as ALS or PD is yet to be investigated. Even though the pre‐clinical results in AD and ageing look promising, the use of senolytic approaches to eliminate senescent cells should be considered cautiously, due to the lack of knowledge regarding the role of senescent cells in neurodegeneration. One alternative to avoid the potential negative effects of eliminating senescent cells is to focus on developing therapies aiming at reducing or eliminating SASP to limit neuroinflammation (Guerrero et al., 2021). Senomorphics or SASP inhibitors can limit senescent cell SASP production by inhibiting NF‐κB, JAK–STAT, mTOR or mitochondrial complex I and IV‐related targets. Because senomorphics do not eliminate senescent cells, continuous treatment with SASP inhibitors would be required to obtain long‐lasting effects, which could also increase off‐target effects associated with the suppression of cytokine secretion by other cells. A better understanding of the role of SASP and senescent cells in CNS diseases and ageing is therefore essential to successfully develop senotherapeutic interventions to limit neuroinflammation and target neurodegeneration (Chaib et al., 2022).

## 7 CONCLUSIONS AND FUTURE PERSPECTIVES

We have attempted to briefly review the vast field of the biological pathways that are affected during ageing and have been implicated in the pathogenesis of neurodegenerative disorders. Even though a common feature of neurodegenerative diseases is the abnormal deposition and mis‐localization of insoluble protein aggregates, different cellular pathways contribute to neuronal loss. Furthermore, these pathways are all affected by ageing, which represents the main common risk factor for most neurodegenerative disorders. Cells in all regions of the CNS are affected by ageing, as indicated by the decline of sensory, motor and cognitive functions with time (Hofer et al., 2003).

In the last few years, the concept of ageing has been changed. Ageing is characterized by decreased functional capacity and increased vulnerability to diseases, disability and death. However, individuals of the same chronological age can differ dramatically in their health status. Ageing should be viewed as a condition caused by the age‐associated accumulation of deficits throughout life (Rockwood et al., 2000). In this context, the genetic alterations present at birth and the ‘noxae’ to which one is exposed during life, which will depend on the individual’s lifestyle, both contribute to the biological ageing process (Salvatore, 2020). To increase longevity and quality of life, biomedical sciences should focus on prevention of ageing. Prevention should start in early life with the analysis of genetic predisposition to specific diseases and with the elimination of lifestyle factors that negatively affect body function. This approach will be critical for the prevention of neurodegenerative disorders and to increase the resilience of neuronal cells to stressors. Moreover, studying how the cellular and molecular changes that occur during ageing render neurons vulnerable to degeneration is fundamental for the development of novel therapeutic approaches. Currently, most efforts to treat neurodegenerative disorders focus on strategies that target the insoluble aggregates of proteins specifically associated with each neurodegenerative disorder. So far, most of the clinical trial results have been disappointing because cognitive function is not restored even when protein aggregates are removed. These results point towards the importance of studying the cellular pathways that contribute to neuronal dysfunction, in order to provide combined therapies to patients.

Neuronal function requires an efficient network of pathways that are strictly connected and interdependent. For instance, synaptic function is affected by the inflammatory microenvironment generated by the ageing glial cells. Furthermore, synaptic transmission requires energy and perturbed mitochondrial function has been associated with ageing and neurodegeneration as the quality control of the cellular components is regulated by energy sensors. Additionally, the lysosomal‐dependent, self‐digestive, processing of damaged proteins and organelles, called autophagy, is important to generate nutrients and energy to maintain essential cellular activities. Defects in autophagy result in intracellular accumulation of protein, contributing to the formation of the insoluble aggregates of protein specifically associated with neurodegenerative disorders. Finally, gene expression translates synaptic activity and alterations in metabolic function into changes in gene expression that can profoundly modify neuronal structure and function.

Different therapeutic strategies have been developed to target these cellular pathways, and the drugs have been evaluated for the treatment of different neurodegenerative disorders. However, the future challenges in drug discovery for neurodegenerative disorders are (i) the detection of the earliest events in the neurodegenerative cascade and (ii) the identification of the pathways responsible for the specific vulnerability of cellular populations in each neurodegenerative disease. A better understanding of these mechanisms is critical in the development of disease‐modifying therapies and to design tailored therapies that can be administered to specific patient populations.

### 7.1 Nomenclature of targets and ligands

Key protein targets and ligands in this article are hyperlinked to corresponding entries in the IUPHAR/BPS Guide to PHARMACOLOGY (http://www.guidetopharmacology.org) and are permanently archived in the Concise Guide to PHARMACOLOGY 2021/22 (Alexander, Christopoulos et al., 2021; Alexander, Fabbro et al., 2021; Alexander, Kelly et al., 2021a, b; Alexander, Mathie et al., 2007).

## AUTHOR CONTRIBUTIONS

**Alerie Guzman de la Fuente:** Conceptualization (equal); resources (equal); writing—original draft (equal). **Silvia Pelucchi:** Conceptualization (equal); writing—original draft (equal). **Jerome Mertens:** Conceptualization (equal); resources (equal); writing—review and editing (equal). **Monica Di Luca:** Resources (equal); writing—review and editing (equal). **Daniela Mauceri:** Conceptualization (equal); resources (equal); writing—original draft (equal). **Elena Marcello:** Conceptualization (equal); resources (supporting); writing—original draft (equal).

## CONFLICT OF INTEREST STATEMENT

All the authors have no conflicts of interest.

## Data Availability

N/A‐review.

## References

[bph16078-bib-0001] Acosta, J. C. , Banito, A. , Wuestefeld, T. , Georgilis, A. , Janich, P. , Morton, J. P. , Athineos, D. , Kang, T. W. , Lasitschka, F. , Andrulis, M. , Pascual, G. , Morris, K. J. , Khan, S. , Jin, H. , Dharmalingam, G. , Snijders, A. P. , Carroll, T. , Capper, D. , Pritchard, C. , … Gil, J. (2013). A complex secretory program orchestrated by the inflammasome controls paracrine senescence. Nature Cell Biology, 15, 978–990. 10.1038/ncb2784 23770676 PMC3732483

[bph16078-bib-0002] Alexander, S. P. , Christopoulos, A. , Davenport, A. P. , Kelly, E. , Mathie, A. , Peters, J. A. , Veale, E. L. , Armstrong, J. F. , Faccenda, E. , Harding, S. D. , Pawson, A. J. , Southan, C. , Davies, J. A. , Abbracchio, M. P. , Alexander, W. , Al‐hosaini, K. , Bäck, M. , Barnes, N. M. , Bathgate, R. , … Ye, R. D. (2021). THE CONCISE GUIDE TO PHARMACOLOGY 2021/22: G protein‐coupled receptors. British Journal of Pharmacology, 178(S1), S27–S156. 10.1111/bph.15538 34529832

[bph16078-bib-0004] Alexander, S. P. , Fabbro, D. , Kelly, E. , Mathie, A. , Peters, J. A. , Veale, E. L. , Armstrong, J. F. , Faccenda, E. , Harding, S. D. , Pawson, A. J. , Southan, C. , Davies, J. A. , Boison, D. , Burns, K. E. , Dessauer, C. , Gertsch, J. , Helsby, N. A. , Izzo, A. A. , Koesling, D. , … Wong, S. S. (2021b). THE CONCISE GUIDE TO PHARMACOLOGY 2021/22: Enzymes. British Journal of Pharmacology, 178(S1), S313–S411. 10.1111/bph.15542 34529828

[bph16078-bib-0005] Alexander, S. P. , Kelly, E. , Mathie, A. , Peters, J. A. , Veale, E. L. , Armstrong, J. F. , Faccenda, E. , Harding, S. D. , Pawson, A. J. , Southan, C. , Buneman, O. P. , Cidlowski, J. A. , Christopoulos, A. , Davenport, A. P. , Fabbro, D. , Spedding, M. , Striessnig, J. , Davies, J. A. , Ahlers‐Dannen, K. E. , … Zolghadri, Y. (2021). THE CONCISE GUIDE TO PHARMACOLOGY 2021/22: Other Protein Targets. British Journal of Pharmacology, 178(S1), S1–S26. 10.1111/bph.15537 34529830 PMC9513948

[bph16078-bib-0006] Alexander, S. P. , Kelly, E. , Mathie, A. , Peters, J. A. , Veale, E. L. , Armstrong, J. F. , Faccenda, E. , Harding, S. D. , Pawson, A. J. , Southan, C. , Davies, J. A. , Amarosi, L. , Anderson, C. M. H. , Beart, P. M. , Broer, S. , Dawson, P. A. , Hagenbuch, B. , Hammond, J. R. , Inui, K.‐I. , … Verri, T. (2021). THE CONCISE GUIDE TO PHARMACOLOGY 2021/22: Transporters. British Journal of Pharmacology, 178(S1), S412–S513. 10.1111/bph.15543 34529826

[bph16078-bib-0007] Alexander, S. P. , Mathie, A. , Peters, J. A. , Veale, E. L. , Striessnig, J. , Kelly, E. , Armstrong, J. F. , Faccenda, E. , Harding, S. D. , Pawson, A. J. , Southan, C. , Davies, J. A. , Aldrich, R. W. , Attali, B. , Baggetta, A. M. , Becirovic, E. , Biel, M. , Bill, R. M. , Catterall, W. A. , … Zhu, M. (2021). THE CONCISE GUIDE TO PHARMACOLOGY 2021/22: Ion channels. British Journal of Pharmacology, 178(S1), S157–S245. 10.1111/bph.15539 34529831

[bph16078-bib-0008] Alkon, D. L. , Sun, M.‐K. , & Nelson, T. J. (2007). PKC signaling deficits: A mechanistic hypothesis for the origins of Alzheimer's disease. Trends in Pharmacological Sciences, 28, 51–60. 10.1016/j.tips.2006.12.002 17218018

[bph16078-bib-0009] Amatniek, J. C. , Hauser, W. A. , DelCastillo‐Castaneda, C. , Jacobs, D. M. , Marder, K. , Bell, K. , Albert, M. , Brandt, J. , & Stern, Y. (2006). Incidence and predictors of seizures in patients with Alzheimer's disease. Epilepsia, 47, 867–872. 10.1111/j.1528-1167.2006.00554.x 16686651

[bph16078-bib-0010] Anderson, K. W. , Chen, J. , Wang, M. , Mast, N. , Pikuleva, I. A. , & Turko, I. v. (2015). Quantification of histone deacetylase isoforms in human frontal cortex, human retina, and mouse brain. PLoS ONE, 10, e0126592. 10.1371/journal.pone.0126592 25962138 PMC4427357

[bph16078-bib-0011] Anoar, S. , Woodling, N. S. , & Niccoli, T. (2021). Mitochondria dysfunction in frontotemporal dementia/amyotrophic lateral sclerosis: Lessons from *drosophila* models. Frontiers in Neuroscience, 15, 786076. 10.3389/fnins.2021.786076 34899176 PMC8652125

[bph16078-bib-0012] Aytan, N. , Choi, J.‐K. , Carreras, I. , Brinkmann, V. , Kowall, N. W. , Jenkins, B. G. , & Dedeoglu, A. (2016). Fingolimod modulates multiple neuroinflammatory markers in a mouse model of Alzheimer's disease. Scientific Reports, 6, 24939. 10.1038/srep24939 27117087 PMC4847010

[bph16078-bib-0013] Baek, H. , Ye, M. , Kang, G.‐H. , Lee, C. , Lee, G. , Choi, D. B. , Jung, J. , Kim, H. , Lee, S. , Kim, J. S. , Lee, H. J. , Shim, I. , Lee, J. H. , & Bae, H. (2016). Neuroprotective effects of CD4^+^CD25^+^Foxp3^+^ regulatory T cells in a 3xTg‐AD Alzheimer's disease model. Oncotarget, 7, 69347–69357. 10.18632/oncotarget.12469 27713140 PMC5342482

[bph16078-bib-0014] Bak, L. K. , Schousboe, A. , & Waagepetersen, H. S. (2006). The glutamate/GABA‐glutamine cycle: Aspects of transport, neurotransmitter homeostasis and ammonia transfer. Journal of Neurochemistry, 98, 641–653. 10.1111/j.1471-4159.2006.03913.x 16787421

[bph16078-bib-0015] Baker, D. J. , Childs, B. G. , Durik, M. , Wijers, M. E. , Sieben, C. J. , Zhong, J. , A. Saltness, R. , Jeganathan, K. B. , Verzosa, G. C. , Pezeshki, A. , Khazaie, K. , Miller, J. D. , & van Deursen, J. M. (2016). Naturally occurring p16^Ink4a^‐positive cells shorten healthy lifespan. Nature, 530, 184–189. 10.1038/nature16932 26840489 PMC4845101

[bph16078-bib-0016] Bartzokis, G. , Beckson, M. , Lu, P. H. , Nuechterlein, K. H. , Edwards, N. , & Mintz, J. (2001). Age‐related changes in frontal and temporal lobe volumes in men: A magnetic resonance imaging study. Archives of General Psychiatry, 58, 461–465. 10.1001/archpsyc.58.5.461 11343525

[bph16078-bib-0017] Baughman, J. M. , Perocchi, F. , Girgis, H. S. , Plovanich, M. , Belcher‐Timme, C. A. , Sancak, Y. , Bao, X. R. , Strittmatter, L. , Goldberger, O. , Bogorad, R. L. , Koteliansky, V. , & Mootha, V. K. (2011). Integrative genomics identifies MCU as an essential component of the mitochondrial calcium uniporter. Nature, 476, 341–345. 10.1038/nature10234 21685886 PMC3486726

[bph16078-bib-0018] Bayraktar, G. , & Kreutz, M. R. (2018). Neuronal DNA methyltransferases: Epigenetic mediators between synaptic activity and gene expression? The Neuroscientist, 24, 171–185. 10.1177/1073858417707457 28513272 PMC5846851

[bph16078-bib-0019] Beers, D. R. , Zhao, W. , Wang, J. , Zhang, X. , Wen, S. , Neal, D. , Thonhoff, J. R. , Alsuliman, A. S. , Shpall, E. J. , Rezvani, K. , & Appel, S. H. (2017). ALS patients' regulatory T lymphocytes are dysfunctional, and correlate with disease progression rate and severity. JCI Insight, 2, e89530. 28289705 10.1172/jci.insight.89530 PMC5333967

[bph16078-bib-0020] Bertram, L. , & Tanzi, R. E. (2005). The genetic epidemiology of neurodegenerative disease. Journal of Clinical Investigation, 115, 1449–1457. 10.1172/JCI24761 15931380 PMC1137006

[bph16078-bib-0021] Blau, C. W. , Cowley, T. R. , O'Sullivan, J. , Grehan, B. , Browne, T. C. , Kelly, L. , Birch, A. , Murphy, N. , Kelly, A. M. , Kerskens, C. M. , & Lynch, M. A. (2012). The age‐related deficit in LTP is associated with changes in perfusion and blood‐brain barrier permeability. Neurobiology of Aging, 33, e23–e35. 10.1016/j.neurobiolaging.2011.09.035 22071124

[bph16078-bib-0022] Brochard, V. , Combadière, B. , Prigent, A. , Laouar, Y. , Perrin, A. , Beray‐Berthat, V. , Bonduelle, O. , Alvarez‐Fischer, D. , Callebert, J. , Launay, J. M. , Duyckaerts, C. , Flavell, R. A. , Hirsch, E. C. , & Hunot, S. (2009). Infiltration of CD4^+^ lymphocytes into the brain contributes to neurodegeneration in a mouse model of Parkinson disease. The Journal of Clinical Investigation, 119, 182–192. 10.1172/JCI36470 19104149 PMC2613467

[bph16078-bib-0023] Bruneteau, G. , Simonet, T. , Bauché, S. , Mandjee, N. , Malfatti, E. , Girard, E. , Tanguy, M. L. , Behin, A. , Khiami, F. , Sariali, E. , Hell‐Remy, C. , Salachas, F. , Pradat, P. F. , Fournier, E. , Lacomblez, L. , Koenig, J. , Romero, N. B. , Fontaine, B. , Meininger, V. , … Hantaï, D. (2013). Muscle histone deacetylase 4 upregulation in amyotrophic lateral sclerosis: Potential role in reinnervation ability and disease progression. Brain, 136, 2359–2368. 10.1093/brain/awt164 23824486

[bph16078-bib-0024] Brynskikh, A. , Warren, T. , Zhu, J. , & Kipnis, J. (2008). Adaptive immunity affects learning behavior in mice. Brain, Behavior, and Immunity, 22, 861–869. 10.1016/j.bbi.2007.12.008 18249087

[bph16078-bib-0025] Bussian, T. J. , Aziz, A. , Meyer, C. F. , Swenson, B. L. , van Deursen, J. M. , & Baker, D. J. (2018). Clearance of senescent glial cells prevents tau‐dependent pathology and cognitive decline. Nature, 562, 578–582. 10.1038/s41586-018-0543-y 30232451 PMC6206507

[bph16078-bib-0026] Carrano, N. , Samaddar, T. , Brunialti, E. , Franchini, L. , Marcello, E. , Ciana, P. , Mauceri, D. , di Luca, M. , & Gardoni, F. (2019). The synaptonuclear messenger RNF10 acts as an architect of neuronal morphology. Molecular Neurobiology, 56, 7583–7593. 10.1007/s12035-019-1631-1 31069631

[bph16078-bib-0027] Castelli, V. , Benedetti, E. , Antonosante, A. , Catanesi, M. , Pitari, G. , Ippoliti, R. , Cimini, A. , & d'Angelo, M. (2019). Neuronal cells rearrangement during aging and neurodegenerative disease: Metabolism, oxidative stress and organelles dynamic. Frontiers in Molecular Neuroscience, 12, 132. 10.3389/fnmol.2019.00132 31191244 PMC6546816

[bph16078-bib-0028] Chaib, S. , Tchkonia, T. , & Kirkland, J. L. (2022). Cellular senescence and senolytics: The path to the clinic. Nature Medicine, 28, 1556–1568. 10.1038/s41591-022-01923-y PMC9599677 35953721

[bph16078-bib-0029] Chang, Y.‐Y. , Juhász, G. , Goraksha‐Hicks, P. , Arsham, A. M. , Mallin, D. R. , Muller, L. K. , & Neufeld, T. P. (2009). Nutrient‐dependent regulation of autophagy through the target of rapamycin pathway. Biochemical Society Transactions, 37, 232–236. 10.1042/BST0370232 19143638

[bph16078-bib-0030] Cheah, B. C. , Vucic, S. , Krishnan, A. , & Kiernan, M. C. (2010). Riluzole, neuroprotection and amyotrophic lateral sclerosis. Current Medicinal Chemistry, 17, 1942–1959. 10.2174/092986710791163939 20377511

[bph16078-bib-0031] Chen, J.‐F. , Liu, K. , Hu, B. , Li, R.‐R. , Xin, W. , Chen, H. , Wang, F. , Chen, L. , Li, R. X. , Ren, S. Y. , Xiao, L. , Chan, J. R. , & Mei, F. (2021). Enhancing myelin renewal reverses cognitive dysfunction in a murine model of Alzheimer's disease. Neuron, 109, 2292–2307.e5. 10.1016/j.neuron.2021.05.012 34102111 PMC8298291

[bph16078-bib-0032] Chia, L. S. , Thompson, J. E. , & Moscarello, M. A. (1983). Changes in lipid phase behaviour in human myelin during maturation and aging. FEBS Letters, 157, 155–158. 10.1016/0014-5793(83)81136-3 6862012

[bph16078-bib-0033] Choi, Y. , Kim, H.‐S. , Shin, K. Y. , Kim, E.‐M. , Kim, M. , Kim, H.‐S. , Park, C. H. , Jeong, Y. H. , Yoo, J. , Lee, J. P. , Chang, K. A. , Kim, S. , & Suh, Y. H. (2007). Minocycline attenuates neuronal cell death and improves cognitive impairment in Alzheimer's disease models. Neuropsychopharmacology, 32, 2393–2404. 10.1038/sj.npp.1301377 17406652

[bph16078-bib-0034] Chouliaras, L. , Mastroeni, D. , Delvaux, E. , Grover, A. , Kenis, G. , Hof, P. R. , Steinbusch, H. W. M. , Coleman, P. D. , Rutten, B. P. F. , & van den Hove, D. L. A. (2013). Consistent decrease in global DNA methylation and hydroxymethylation in the hippocampus of Alzheimer's disease patients. Neurobiology of Aging, 34, 2091–2099. 10.1016/j.neurobiolaging.2013.02.021 23582657 PMC3955118

[bph16078-bib-0035] Chu, C. T. (2010). A pivotal role for PINK1 and autophagy in mitochondrial quality control: Implications for Parkinson disease. Human Molecular Genetics, 19, R28–R37. 10.1093/hmg/ddq143 20385539 PMC2875056

[bph16078-bib-0036] Chu, T.‐T. , Gao, N. , Li, Q.‐Q. , Chen, P.‐G. , Yang, X.‐F. , Chen, Y.‐X. , Zhao, Y. F. , & Li, Y. M. (2016). Specific knockdown of endogenous tau protein by peptide‐directed ubiquitin‐proteasome degradation. Cell Chemical Biology, 23, 453–461. 10.1016/j.chembiol.2016.02.016 27105281

[bph16078-bib-0037] Chuquet, J. , Quilichini, P. , Nimchinsky, E. A. , & Buzsaki, G. (2010). Predominant enhancement of glucose uptake in astrocytes versus neurons during activation of the somatosensory cortex. Journal of Neuroscience, 30, 15298–15303. 10.1523/JNEUROSCI.0762-10.2010 21068334 PMC2997269

[bph16078-bib-0038] Citri, A. , & Malenka, R. C. (2008). Synaptic plasticity: Multiple forms, functions, and mechanisms. Neuropsychopharmacology, 33, 18–41. 10.1038/sj.npp.1301559 17728696

[bph16078-bib-0039] Coppieters, N. , Dieriks, B. v. , Lill, C. , Faull, R. L. M. , Curtis, M. A. , & Dragunow, M. (2014). Global changes in DNA methylation and hydroxymethylation in Alzheimer's disease human brain. Neurobiology of Aging, 35, 1334–1344. 10.1016/j.neurobiolaging.2013.11.031 24387984

[bph16078-bib-0040] Coque, E. , Salsac, C. , Espinosa‐Carrasco, G. , Varga, B. , Degauque, N. , Cadoux, M. , Crabé, R. , Virenque, A. , Soulard, C. , Fierle, J. K. , Brodovitch, A. , Libralato, M. , Végh, A. G. , Venteo, S. , Scamps, F. , Boucraut, J. , Laplaud, D. , Hernandez, J. , Gergely, C. , … Raoul, C. (2019). Cytotoxic CD8^+^ T lymphocytes expressing ALS‐causing SOD1 mutant selectively trigger death of spinal motoneurons. Proceedings of the National Academy of Sciences, 116, 2312–2317. 10.1073/pnas.1815961116 PMC6369778 30674678

[bph16078-bib-0041] Cudkowicz, M. (2010). A futility study of minocycline in Huntington's disease. Movement Disorders, 25, 2219–2224. 10.1002/mds.23236 20721920 PMC8801051

[bph16078-bib-0042] Cummings, J. , Lee, G. , Nahed, P. , Kambar, M. E. Z. N. , Zhong, K. , Fonseca, J. , & Taghva, K. (2022). Alzheimer's disease drug development pipeline: 2022. Alzheimer's Dement, 8, e12295. 10.1002/trc2.12295 PMC9066743 35516416

[bph16078-bib-0043] Dai, W. , Wang, G. , Chwa, J. , Oh, M. E. , Abeywardana, T. , Yang, Y. , Wang, Q. A. , & Jiang, L. (2020). Mitochondrial division inhibitor (mdivi‐1) decreases oxidative metabolism in cancer. British Journal of Cancer, 122, 1288–1297. 10.1038/s41416-020-0778-x 32147668 PMC7188673

[bph16078-bib-0044] Daniele, S. , Giacomelli, C. , & Martini, C. (2018). Brain ageing and neurodegenerative disease: The role of cellular waste management. Biochemical Pharmacology, 158, 207–216. 10.1016/j.bcp.2018.10.030 30393045

[bph16078-bib-0045] Dansokho, C. , Ait Ahmed, D. , Aid, S. , Toly‐Ndour, C. , Chaigneau, T. , Calle, V. , Cagnard, N. , Holzenberger, M. , Piaggio, E. , Aucouturier, P. , & Dorothée, G. (2016). Regulatory T cells delay disease progression in Alzheimer‐like pathology. Brain, 139, 1237–1251. 10.1093/brain/awv408 26912648

[bph16078-bib-0046] David, D. C. , Hauptmann, S. , Scherping, I. , Schuessel, K. , Keil, U. , Rizzu, P. , Ravid, R. , Dröse, S. , Brandt, U. , Müller, W. E. , Eckert, A. , & Götz, J. (2005). Proteomic and functional analyses reveal a mitochondrial dysfunction in P301L tau transgenic mice. Journal of Biological Chemistry, 280, 23802–23814. 10.1074/jbc.M500356200 15831501

[bph16078-bib-0047] Dehay, B. , Bové, J. , Rodríguez‐Muela, N. , Perier, C. , Recasens, A. , Boya, P. , & Vila, M. (2010). Pathogenic lysosomal depletion in Parkinson's disease. Journal of Neuroscience, 30, 12535–12544. 10.1523/JNEUROSCI.1920-10.2010 20844148 PMC6633458

[bph16078-bib-0048] Dharmadasa, T. , Henderson, R. D. , Talman, P. S. , Macdonell, R. A. L. , Mathers, S. , Schultz, D. W. , Needham, M. , Zoing, M. , Vucic, S. , & Kiernan, M. C. (2017). Motor neurone disease: Progress and challenges. Medical Journal of Australia, 206, 357–362. 10.5694/mja16.01063 28446118

[bph16078-bib-0049] di Pardo, A. , Amico, E. , Favellato, M. , Castrataro, R. , Fucile, S. , Squitieri, F. , & Maglione, V. (2014). FTY720 (fingolimod) is a neuroprotective and disease‐modifying agent in cellular and mouse models of Huntington disease. Human Molecular Genetics, 23, 2251–2265. 10.1093/hmg/ddt615 24301680

[bph16078-bib-0050] Dickstein, D. L. , Kabaso, D. , Rocher, A. B. , Luebke, J. I. , Wearne, S. L. , & Hof, P. R. (2007). Changes in the structural complexity of the aged brain. Aging Cell, 6, 275–284. 10.1111/j.1474-9726.2007.00289.x 17465981 PMC2441530

[bph16078-bib-0051] Dieterich, D. C. , Karpova, A. , Mikhaylova, M. , Zdobnova, I. , König, I. , Landwehr, M. , Kreutz, M. , Smalla, K. H. , Richter, K. , Landgraf, P. , Reissner, C. , Boeckers, T. M. , Zuschratter, W. , Spilker, C. , Seidenbecher, C. I. , Garner, C. C. , Gundelfinger, E. D. , & Kreutz, M. R. (2008). Caldendrin–Jacob: A protein liaison that couples NMDA receptor signalling to the nucleus. PLoS Biology, 6, e34. 10.1371/journal.pbio.0060034 18303947 PMC2253627

[bph16078-bib-0052] DiLuca, M. , & Olesen, J. (2014). The cost of brain diseases: A burden or a challenge? Neuron, 82, 1205–1208. 10.1016/j.neuron.2014.05.044 24945765

[bph16078-bib-0053] Dinamarca, M. C. , Guzzetti, F. , Karpova, A. , Lim, D. , Mitro, N. , Musardo, S. , Mellone, M. , Marcello, E. , Stanic, J. , Samaddar, T. , Burguière, A. , Caldarelli, A. , Genazzani, A. A. , Perroy, J. , Fagni, L. , Canonico, P. L. , Kreutz, M. R. , Gardoni, F. , & Luca, M. D. (2016). Ring finger protein 10 is a novel synaptonuclear messenger encoding activation of NMDA receptors in hippocampus. eLife, 5, e12430. 10.7554/eLife.12430 26977767 PMC4805553

[bph16078-bib-0054] Dulken, B. W. , Buckley, M. T. , Navarro Negredo, P. , Saligrama, N. , Cayrol, R. , Leeman, D. S. , George, B. M. , Boutet, S. C. , Hebestreit, K. , Pluvinage, J. V. , Wyss‐Coray, T. , Weissman, I. L. , Vogel, H. , Davis, M. M. , & Brunet, A. (2019). Single‐cell analysis reveals T cell infiltration in old neurogenic niches. Nature, 571, 205–210. 10.1038/s41586-019-1362-5 31270459 PMC7111535

[bph16078-bib-0055] Dumont, M. , Kipiani, K. , Yu, F. , Wille, E. , Katz, M. , Calingasan, N. Y. , Gouras, G. K. , Lin, M. T. , & Beal, M. F. (2011). Coenzyme Q10 decreases amyloid pathology and improves behavior in a transgenic mouse model of Alzheimer's disease. Journal of Alzheimer's Disease, 27, 211–223. 10.3233/JAD-2011-110209 PMC3267988 21799249

[bph16078-bib-0056] Ettle, B. , Kerman, B. E. , Valera, E. , Gillmann, C. , Schlachetzki, J. C. M. , Reiprich, S. , Büttner, C. , Ekici, A. B. , Reis, A. , Wegner, M. , Bäuerle, T. , Riemenschneider, M. J. , Masliah, E. , Gage, F. H. , & Winkler, J. (2016). α‐Synuclein‐induced myelination deficit defines a novel interventional target for multiple system atrophy. Acta Neuropathologica, 132, 59–75. 10.1007/s00401-016-1572-y 27059609 PMC4912450

[bph16078-bib-0057] Fainzilber, M. , Budnik, V. , Segal, R. A. , & Kreutz, M. R. (2011). From synapse to nucleus and back again—Communication over distance within neurons. Journal of Neuroscience, 31, 16045–16048. 10.1523/JNEUROSCI.4006-11.2011 22072654 PMC3242373

[bph16078-bib-0058] Fan, S.‐J. , Huang, F.‐I. , Liou, J.‐P. , & Yang, C.‐R. (2018). The novel histone de acetylase 6 inhibitor, MPT0G211, ameliorates tau phosphorylation and cognitive deficits in an Alzheimer's disease model. Cell Death & Disease, 9, 655. 10.1038/s41419-018-0688-5 29844403 PMC5974403

[bph16078-bib-0059] Faridar, A. , Vasquez, M. , Thome, A. D. , Yin, Z. , Xuan, H. , Wang, J. H. , Wen, S. , Li, X. , Thonhoff, J. R. , Zhao, W. , Zhao, H. , Beers, D. R. , Wong, S. T. C. , Masdeu, J. C. , & Appel, S. H. (2022). Ex vivo expanded human regulatory T cells modify neuroinflammation in a preclinical model of Alzheimer's disease. Acta Neuropathologica Communications, 10, 144. 10.1186/s40478-022-01447-z 36180898 PMC9524037

[bph16078-bib-0060] Filippone, A. , Esposito, E. , Mannino, D. , Lyssenko, N. , & Praticò, D. (2022). The contribution of altered neuronal autophagy to neurodegeneration. Pharmacology & Therapeutics, 238, 108178. 10.1016/j.pharmthera.2022.108178 35351465 PMC9510148

[bph16078-bib-0061] Forman, M. S. , Trojanowski, J. Q. , & Lee, V. M.‐Y. (2004). Neurodegenerative diseases: A decade of discoveries paves the way for therapeutic breakthroughs. Nature Medicine, 10, 1055–1063. 10.1038/nm1113 15459709

[bph16078-bib-0062] Gao, J. , Wang, L. , Yan, T. , Perry, G. , & Wang, X. (2019). TDP‐43 proteinopathy and mitochondrial abnormalities in neurodegeneration. Molecular and Cellular Neuroscience, 100, 103396. 10.1016/j.mcn.2019.103396 31445085 PMC6874890

[bph16078-bib-0063] Garofalo, S. , Cocozza, G. , Bernardini, G. , Savage, J. , Raspa, M. , Aronica, E. , Tremblay, M. E. , Ransohoff, R. M. , Santoni, A. , & Limatola, C. (2022). Blocking immune cell infiltration of the central nervous system to tame neuroinflammation in amyotrophic lateral sclerosis. Brain, Behavior, and Immunity, 105, 1–14. 10.1016/j.bbi.2022.06.004 35688338

[bph16078-bib-0064] Gate, D. , Saligrama, N. , Leventhal, O. , Yang, A. C. , Unger, M. S. , Middeldorp, J. , Chen, K. , Lehallier, B. , Channappa, D. , de Los Santos, M. B. , McBride, A. , Pluvinage, J. , Elahi, F. , Tam, G. K. Y. , Kim, Y. , Greicius, M. , Wagner, A. D. , Aigner, L. , Galasko, D. R. , … Wyss‐Coray, T. (2020). Clonally expanded CD8 T cells patrol the cerebrospinal fluid in Alzheimer's disease. Nature, 577, 399–404. 10.1038/s41586-019-1895-7 31915375 PMC7445078

[bph16078-bib-0065] Gelon, P. A. , Dutchak, P. A. , & Sephton, C. F. (2022). Synaptic dysfunction in ALS and FTD: Anatomical and molecular changes provide insights into mechanisms of disease. Frontiers in Molecular Neuroscience, 15, 1000183. 10.3389/fnmol.2022.1000183 36263379 PMC9575515

[bph16078-bib-0066] Gómez‐Suaga, P. , Luzón‐Toro, B. , Churamani, D. , Zhang, L. , Bloor‐Young, D. , Patel, S. , Woodman, P. G. , Churchill, G. C. , & Hilfiker, S. (2012). Leucine‐rich repeat kinase 2 regulates autophagy through a calcium‐dependent pathway involving NAADP. Human Molecular Genetics, 21, 511–525. 10.1093/hmg/ddr481 22012985 PMC3259011

[bph16078-bib-0067] Gordon, P. H. , Moore, D. H. , Miller, R. G. , Florence, J. M. , Verheijde, J. L. , Doorish, C. , Hilton, J. F. , Spitalny, G. M. , MacArthur, R. , Mitsumoto, H. , Neville, H. E. , Boylan, K. , Mozaffar, T. , Belsh, J. M. , Ravits, J. , Bedlack, R. S. , Graves, M. C. , McCluskey, L. , Barohn, R. J. , … Western ALS Study Group . (2007). Efficacy of minocycline in patients with amyotrophic lateral sclerosis: A phase III randomised trial. Lancet Neurology, 6, 1045–1053. 10.1016/S1474-4422(07)70270-3 17980667

[bph16078-bib-0068] Gräff, J. , Rei, D. , Guan, J.‐S. , Wang, W.‐Y. , Seo, J. , Hennig, K. M. , Nieland, T. J. F. , Fass, D. M. , Kao, P. F. , Kahn, M. , Su, S. C. , Samiei, A. , Joseph, N. , Haggarty, S. J. , Delalle, I. , & Tsai, L. H. (2012). An epigenetic blockade of cognitive functions in the neurodegenerating brain. Nature, 483, 222–226. 10.1038/nature10849 22388814 PMC3498952

[bph16078-bib-0069] Green, A. J. , Gelfand, J. M. , Cree, B. A. , Bevan, C. , Boscardin, W. J. , Mei, F. , Inman, J. , Arnow, S. , Devereux, M. , Abounasr, A. , Nobuta, H. , Zhu, A. , Friessen, M. , Gerona, R. , von Büdingen, H. C. , Henry, R. G. , Hauser, S. L. , & Chan, J. R. (2017). Clemastine fumarate as a remyelinating therapy for multiple sclerosis (ReBUILD): A randomised, controlled, double‐blind, crossover trial. The Lancet, 390, 2481–2489. 10.1016/S0140-6736(17)32346-2 29029896

[bph16078-bib-0070] Grimm, A. , & Eckert, A. (2017). Brain aging and neurodegeneration: From a mitochondrial point of view. Journal of Neurochemistry, 143, 418–431. 10.1111/jnc.14037 28397282 PMC5724505

[bph16078-bib-0071] Groh, J. , Knöpper, K. , Arampatzi, P. , Yuan, X. , Lößlein, L. , Saliba, A.‐E. , Kastenmüller, W. , & Martini, R. (2021). Accumulation of cytotoxic T cells in the aged CNS leads to axon degeneration and contributes to cognitive and motor decline. Nat Aging, 1, 357–367. 10.1038/s43587-021-00049-z 37117598

[bph16078-bib-0072] Guerrero, A. , de Strooper, B. , & Arancibia‐Cárcamo, I. L. (2021). Cellular senescence at the crossroads of inflammation and Alzheimer's disease. Trends in Neurosciences, 44, 714–727. 10.1016/j.tins.2021.06.007 34366147

[bph16078-bib-0073] Guo, F. , Liu, X. , Cai, H. , & Le, W. (2018). Autophagy in neurodegenerative diseases: Pathogenesis and therapy. Brain Pathology, 28, 3–13. 10.1111/bpa.12545 28703923 PMC5739982

[bph16078-bib-0074] Gupta, R. , Ambasta, R. K. , & Kumar, P. (2020). Pharmacological intervention of histone deacetylase enzymes in the neurodegenerative disorders. Life Sciences, 243, 117278. 10.1016/j.lfs.2020.117278 31926248

[bph16078-bib-0075] Hasel, P. , Rose, I. V. L. , Sadick, J. S. , Kim, R. D. , & Liddelow, S. A. (2021). Neuroinflammatory astrocyte subtypes in the mouse brain. Nature Neuroscience, 24, 1475–1487. 10.1038/s41593-021-00905-6 34413515

[bph16078-bib-0076] Hebert, L. E. , Bienias, J. L. , Aggarwal, N. T. , Wilson, R. S. , Bennett, D. A. , Shah, R. C. , & Evans, D. A. (2010). Change in risk of Alzheimer disease over time. Neurology, 75, 786–791. 10.1212/WNL.0b013e3181f0754f 20805524 PMC2938969

[bph16078-bib-0077] Henderson‐Smith, A. , Fisch, K. M. , Hua, J. , Liu, G. , Ricciardelli, E. , Jepsen, K. , Huentelman, M. , Stalberg, G. , Edland, S. D. , Scherzer, C. R. , Dunckley, T. , & Desplats, P. (2019). DNA methylation changes associated with Parkinson's disease progression: Outcomes from the first longitudinal genome‐wide methylation analysis in blood. Epigenetics, 14, 365–382. 10.1080/15592294.2019.1588682 30871403 PMC6557551

[bph16078-bib-0078] Hill, R. A. , Li, A. M. , & Grutzendler, J. (2018). Lifelong cortical myelin plasticity and age‐related degeneration in the live mammalian brain. Nature Neuroscience, 21, 683–695. 10.1038/s41593-018-0120-6 29556031 PMC5920745

[bph16078-bib-0079] Hofer, S. M. , Berg, S. , & Era, P. (2003). Evaluating the interdependence of aging‐related changes in visual and auditory acuity, balance, and cognitive functioning. Psychology and Aging, 18, 285–305. 10.1037/0882-7974.18.2.285 12825777

[bph16078-bib-0080] Hongpaisan, J. , Sun, M.‐K. , & Alkon, D. L. (2011). PKC ε activation prevents synaptic loss, Aβ elevation, and cognitive deficits in Alzheimer's disease transgenic mice. Journal of Neuroscience, 31, 630–643. 10.1523/JNEUROSCI.5209-10.2011 21228172 PMC6623433

[bph16078-bib-0081] Hou, Y. , Dan, X. , Babbar, M. , Wei, Y. , Hasselbalch, S. G. , Croteau, D. L. , & Bohr, V. A. (2019). Ageing as a risk factor for neurodegenerative disease. Nature Reviews. Neurology, 15, 565–581. 10.1038/s41582-019-0244-7 31501588

[bph16078-bib-0082] Howard, R. , Zubko, O. , Bradley, R. , Harper, E. , Pank, L. , O'Brien, J. , Fox, C. , Tabet, N. , Livingston, G. , Bentham, P. , McShane, R. , Burns, A. , Ritchie, C. , Reeves, S. , Lovestone, S. , Ballard, C. , Noble, W. , Nilforooshan, R. , Wilcock, G. , … for the Minocycline in Alzheimer Disease Efficacy (MADE) Trialist Group . (2020). Minocycline at 2 different dosages vs placebo for patients with mild Alzheimer disease: A randomized clinical trial. JAMA Neurology, 77, 164–174. 10.1001/jamaneurol.2019.3762 31738372 PMC6865324

[bph16078-bib-0083] Hughes, E. G. , Orthmann‐Murphy, J. L. , Langseth, A. J. , & Bergles, D. E. (2018). Myelin remodeling through experience‐dependent oligodendrogenesis in the adult somatosensory cortex. Nature Neuroscience, 21, 696–706. 10.1038/s41593-018-0121-5 29556025 PMC5920726

[bph16078-bib-0084] Hunot, S. , Brugg, B. , Ricard, D. , Michel, P. P. , Muriel, M.‐P. , Ruberg, M. , Faucheux, B. A. , Agid, Y. , & Hirsch, E. C. (1997). Nuclear translocation of NF‐κB is increased in dopaminergic neurons of patients with Parkinson disease. Proceedings of the National Academy of Sciences, 94, 7531–7536. 10.1073/pnas.94.14.7531 PMC23856 9207126

[bph16078-bib-0085] Jancic, D. , Lopez de Armentia, M. , Valor, L. M. , Olivares, R. , & Barco, A. (2009). Inhibition of cAMP response element‐binding protein reduces neuronal excitability and plasticity, and triggers neurodegeneration. Cerebral Cortex, 19, 2535–2547. 10.1093/cercor/bhp004 19213815

[bph16078-bib-0086] Janssen, C. , Schmalbach, S. , Boeselt, S. , Sarlette, A. , Dengler, R. , & Petri, S. (2010). Differential histone deacetylase mRNA expression patterns in amyotrophic lateral sclerosis. Journal of Neuropathology and Experimental Neurology, 69, 573–581. 10.1097/NEN.0b013e3181ddd404 20467334

[bph16078-bib-0215] Ji, C. H. , Kim, H. Y. , Lee, M. J. , Heo, A. J. , Park, D. Y. , Lim, S. , Shin, S. , Ganipisetti, S. , Yang, W. S. , Jung, C. A. , Kim, K. Y. , Jeong, E. H. , Park, S. H. , Bin Kim, S. , Lee, S. J. , Na, J. E. , Kang, J. I. , Chi, H. M. , Kim, H. T. , … Kwon, Y. T. (2022). The AUTOTAC chemical biology platform for targeted protein degradation via the autophagy‐lysosome system. Nature Communications, 13(1), 904. 10.1038/s41467-022-28520-4 PMC8850458 35173167

[bph16078-bib-0087] Johri, A. (2021). Disentangling mitochondria in Alzheimer's disease. International Journal of Molecular Sciences, 22, 11520. 10.3390/ijms222111520 34768950 PMC8583788

[bph16078-bib-0088] Jones, M. K. , Nair, A. , & Gupta, M. (2019). Mast cells in neurodegenerative disease. Frontiers in Cellular Neuroscience, 13, 171. 10.3389/fncel.2019.00171 31133804 PMC6524694

[bph16078-bib-0089] Joshi, P. , Gabrielli, M. , Ponzoni, L. , Pelucchi, S. , Stravalaci, M. , Beeg, M. , Mazzitelli, S. , Braida, D. , Sala, M. , Boda, E. , Buffo, A. , Gobbi, M. , Gardoni, F. , Matteoli, M. , Marcello, E. , & Verderio, C. (2017). Fingolimod limits acute Aβ neurotoxicity and promotes synaptic versus extrasynaptic NMDA receptor functionality in hippocampal neurons. Scientific Reports, 7, 41734. 10.1038/srep41734 28134307 PMC5278353

[bph16078-bib-0090] Kaltschmidt, B. , Uherek, M. , Wellmann, H. , Volk, B. , & Kaltschmidt, C. (1999). Inhibition of NF‐κB potentiates amyloid β‐mediated neuronal apoptosis. Proceedings of the National Academy of Sciences, 96, 9409–9414. 10.1073/pnas.96.16.9409 PMC17796 10430956

[bph16078-bib-0091] Karpova, A. , Mikhaylova, M. , Bera, S. , Bär, J. , Reddy, P. P. , Behnisch, T. , Rankovic, V. , Spilker, C. , Bethge, P. , Sahin, J. , Kaushik, R. , Zuschratter, W. , Kähne, T. , Naumann, M. , Gundelfinger, E. D. , & Kreutz, M. R. (2013). Encoding and transducing the synaptic or extrasynaptic origin of NMDA receptor signals to the nucleus. Cell, 152, 1119–1133. 10.1016/j.cell.2013.02.002 23452857

[bph16078-bib-0092] Kaya, T. , Mattugini, N. , Liu, L. , Ji, H. , Cantuti‐Castelvetri, L. , Wu, J. , Schifferer, M. , Groh, J. , Martini, R. , Besson‐Girard, S. , Kaji, S. , Liesz, A. , Gokce, O. , & Simons, M. (2022). CD8^+^ T cells induce interferon‐responsive oligodendrocytes and microglia in white matter aging. Nature Neuroscience, 25, 1446–1457. 10.1038/s41593-022-01183-6 36280798 PMC9630119

[bph16078-bib-0093] Kenigsbuch, M. , Bost, P. , Halevi, S. , Chang, Y. , Chen, S. , Ma, Q. , Hajbi, R. , Schwikowski, B. , Bodenmiller, B. , Fu, H. , Schwartz, M. , & Amit, I. (2022). A shared disease‐associated oligodendrocyte signature among multiple CNS pathologies. Nature Neuroscience, 25, 876–886. 10.1038/s41593-022-01104-7 35760863 PMC9724210

[bph16078-bib-0094] Keshavarz, M. , Xie, K. , Schaaf, K. , Bano, D. , & Ehninger, D. (2023). Targeting the “hallmarks of aging” to slow aging and treat age‐related disease: Fact or fiction? Molecular Psychiatry, 28, 242–255. 10.1038/s41380-022-01680-x 35840801 PMC9812785

[bph16078-bib-0095] Kim, H. , Han, S. H. , Quan, H. Y. , Jung, Y.‐J. , An, J. , Kang, P. , Park, J. B. , Yoon, B. J. , Seol, G. H. , & Min, S. S. (2012). Bryostatin‐1 promotes long‐term potentiation via activation of PKCα and PKCε in the hippocampus. Neuroscience, 226, 348–355. 10.1016/j.neuroscience.2012.08.055 22986161

[bph16078-bib-0096] Kim, Y. , Zheng, X. , Ansari, Z. , Bunnell, M. C. , Herdy, J. R. , Traxler, L. , Lee, H. , Paquola, A. C. M. , Blithikioti, C. , Ku, M. , Schlachetzki, J. C. M. , Winkler, J. , Edenhofer, F. , Glass, C. K. , Paucar, A. A. , Jaeger, B. N. , Pham, S. , Boyer, L. , Campbell, B. C. , … Gage, F. H. (2018). Mitochondrial aging defects emerge in directly reprogrammed human neurons due to their metabolic profile. Cell Reports, 23, 2550–2558. 10.1016/j.celrep.2018.04.105 29847787 PMC6478017

[bph16078-bib-0097] Koshiba, T. , Detmer, S. A. , Kaiser, J. T. , Chen, H. , McCaffery, J. M. , & Chan, D. C. (2004). Structural basis of mitochondrial tethering by mitofusin complexes. Science, 305, 858–862. 15297672 10.1126/science.1099793

[bph16078-bib-0098] Kriz, J. , Nguyen, M. D. , & Julien, J.‐P. (2002). Minocycline slows disease progression in a mouse model of amyotrophic lateral sclerosis. Neurobiology of Disease, 10, 268–278. 10.1006/nbdi.2002.0487 12270689

[bph16078-bib-0099] Kuruva, C. S. , Manczak, M. , Yin, X. , Ogunmokun, G. , Reddy, A. P. , & Reddy, P. H. (2017). Aqua‐soluble DDQ reduces the levels of Drp1 and Aβ and inhibits abnormal interactions between Aβ and Drp1 and protects Alzheimer's disease neurons from Aβ‐ and Drp1‐induced mitochondrial and synaptic toxicities. Human Molecular Genetics, 26, 3375–3395. 10.1093/hmg/ddx226 28854701 PMC5886305

[bph16078-bib-0100] Lashley, T. , Gami, P. , Valizadeh, N. , Li, A. , Revesz, T. , & Balazs, R. (2015). Alterations in global DNA methylation and hydroxymethylation are not detected in Alzheimer's disease. Neuropathology and Applied Neurobiology, 41, 497–506. 10.1111/nan.12183 25201696 PMC4879505

[bph16078-bib-0101] Laurent, C. , Dorothée, G. , Hunot, S. , Martin, E. , Monnet, Y. , Duchamp, M. , Dong, Y. , Légeron, F. P. , Leboucher, A. , Burnouf, S. , Faivre, E. , Carvalho, K. , Caillierez, R. , Zommer, N. , Demeyer, D. , Jouy, N. , Sazdovitch, V. , Schraen‐Maschke, S. , Delarasse, C. , … Blum, D. (2017). Hippocampal T cell infiltration promotes neuroinflammation and cognitive decline in a mouse model of tauopathy. Brain, 140, 184–200. 10.1093/brain/aww270 27818384 PMC5382942

[bph16078-bib-0102] Lee, H.‐Y. , Fan, S.‐J. , Huang, F.‐I. , Chao, H.‐Y. , Hsu, K.‐C. , Lin, T. E. , Yeh, T. K. , Lai, M. J. , Li, Y. H. , Huang, H. L. , Yang, C. R. , & Liou, J. P. (2018). 5‐Aroylindoles act as selective histone deacetylase 6 inhibitors ameliorating Alzheimer's disease phenotypes. Journal of Medicinal Chemistry, 61, 7087–7102. 10.1021/acs.jmedchem.8b00151 30028616

[bph16078-bib-0103] Lee, J. V. , Carrer, A. , Shah, S. , Snyder, N. W. , Wei, S. , Venneti, S. , Worth, A. J. , Yuan, Z. F. , Lim, H. W. , Liu, S. , Jackson, E. , Aiello, N. M. , Haas, N. B. , Rebbeck, T. R. , Judkins, A. , Won, K. J. , Chodosh, L. A. , Garcia, B. A. , Stanger, B. Z. , … Wellen, K. E. (2014). Akt‐dependent metabolic reprogramming regulates tumor cell histone acetylation. Cell Metabolism, 20, 306–319. 10.1016/j.cmet.2014.06.004 24998913 PMC4151270

[bph16078-bib-0104] Li, S. , & Selkoe, D. J. (2020). A mechanistic hypothesis for the impairment of synaptic plasticity by soluble Aβ oligomers from Alzheimer's brain. Journal of Neurochemistry, 154, 583–597. 10.1111/jnc.15007 32180217 PMC7487043

[bph16078-bib-0105] Li, T. , Martin, E. , Abada, Y. , Boucher, C. , Cès, A. , Youssef, I. , Fenaux, G. , Forand, Y. , Legrand, A. , Nachiket, N. , Dhenain, M. , Hermine, O. , Dubreuil, P. , Delarasse, C. , & Delatour, B. (2020). Effects of chronic masitinib treatment in APPswe/PSEN1dE9 transgenic mice modeling Alzheimer's disease. Journal of Alzheimer's Disease, 76, 1339–1345. 10.3233/JAD-200466 32623401

[bph16078-bib-0106] Li, X. , Egervari, G. , Wang, Y. , Berger, S. L. , & Lu, Z. (2018). Regulation of chromatin and gene expression by metabolic enzymes and metabolites. Nature Reviews. Molecular Cell Biology, 19, 563–578. 10.1038/s41580-018-0029-7 29930302 PMC6907087

[bph16078-bib-0107] Liang, J. , Shao, S. H. , Xu, Z.‐X. , Hennessy, B. , Ding, Z. , Larrea, M. , Kondo, S. , Dumont, D. J. , Gutterman, J. U. , Walker, C. L. , Slingerland, J. M. , & Mills, G. B. (2007). The energy sensing LKB1–AMPK pathway regulates p27kip1 phosphorylation mediating the decision to enter autophagy or apoptosis. Nature Cell Biology, 9, 218–224. 10.1038/ncb1537 17237771

[bph16078-bib-0108] Liston, A. , Dooley, J. , & Yshii, L. (2022). Brain‐resident regulatory T cells and their role in health and disease. Immunology Letters, 248, 26–30. 10.1016/j.imlet.2022.06.005 35697195

[bph16078-bib-0109] Litke, C. , Hagenston, A. M. , Kenkel, A.‐K. , Paldy, E. , Lu, J. , Kuner, R. , & Mauceri, D. (2022). Organic anion transporter 1 is an HDAC4‐regulated mediator of nociceptive hypersensitivity in mice. Nature Communications, 13, 875. 10.1038/s41467-022-28357-x PMC8847565 35169129

[bph16078-bib-0110] Liu, H.‐H. , & Jan, Y.‐N. (2020). Mechanisms of neurite repair. Current Opinion in Neurobiology, 63, 53–58. 10.1016/j.conb.2020.02.010 32278210 PMC7534396

[bph16078-bib-0111] Lucin, K. M. , O'Brien, C. E. , Bieri, G. , Czirr, E. , Mosher, K. I. , Abbey, R. J. , Mastroeni, D. F. , Rogers, J. , Spencer, B. , Masliah, E. , & Wyss‐Coray, T. (2013). Microglial beclin 1 regulates retromer trafficking and phagocytosis and is impaired in Alzheimer's disease. Neuron, 79, 873–886. 10.1016/j.neuron.2013.06.046 24012002 PMC3779465

[bph16078-bib-0112] Lundgren, J. L. , Vandermeulen, L. , Sandebring‐Matton, A. , Ahmed, S. , Winblad, B. , di Luca, M. , Tjernberg, L. O. , Marcello, E. , & Frykman, S. (2020). Proximity ligation assay reveals both pre‐ and postsynaptic localization of the APP‐processing enzymes ADAM10 and BACE1 in rat and human adult brain. BMC Neuroscience, 21, 6. 10.1186/s12868-020-0554-0 32019490 PMC7001251

[bph16078-bib-0113] Lyseng‐Williamson, K. A. (2011). Levetiracetam: A review of its use in epilepsy. Drugs, 71, 489–514. 10.2165/11204490-000000000-00000 21395360

[bph16078-bib-0114] Malinverno, M. , Carta, M. , Epis, R. , Marcello, E. , Verpelli, C. , Cattabeni, F. , Sala, C. , Mulle, C. , di Luca, M. , & Gardoni, F. (2010). Synaptic localization and activity of ADAM10 regulate excitatory synapses through N‐cadherin cleavage. The Journal of Neuroscience, 30, 16343–16355. 10.1523/JNEUROSCI.1984-10.2010 21123580 PMC6634827

[bph16078-bib-0115] Malpartida, A. B. , Williamson, M. , Narendra, D. P. , Wade‐Martins, R. , & Ryan, B. J. (2021). Mitochondrial dysfunction and mitophagy in Parkinson's disease: From mechanism to therapy. Trends in Biochemical Sciences, 46, 329–343. 10.1016/j.tibs.2020.11.007 33323315

[bph16078-bib-0116] Manczak, M. , Kandimalla, R. , Yin, X. , & Reddy, P. H. (2019). Mitochondrial division inhibitor 1 reduces dynamin‐related protein 1 and mitochondrial fission activity. Human Molecular Genetics, 28, 177–199. 10.1093/hmg/ddy335 30239719 PMC6322070

[bph16078-bib-0117] Mandrioli, J. , D'Amico, R. , Zucchi, E. , Gessani, A. , Fini, N. , Fasano, A. , Caponnetto, C. , Chiò, A. , Dalla Bella, E. , Lunetta, C. , Mazzini, L. , Marinou, K. , Sorarù, G. , de Biasi, S. , Lo Tartaro, D. , Pinti, M. , Cossarizza, A. , & RAP‐ALS investigators group . (2018). Rapamycin treatment for amyotrophic lateral sclerosis: Protocol for a phase II randomized, double‐blind, placebo‐controlled, multicenter, clinical trial (RAP‐ALS trial). Medicine, 97, e11119. 10.1097/MD.0000000000011119 29901635 PMC6024184

[bph16078-bib-0118] Manocha, G. , Ghatak, A. , Puig, K. , & Combs, C. (2018). Anti‐α4β1 integrin antibodies attenuated brain inflammatory changes in a mouse model of Alzheimer's disease. Current Alzheimer Research, 15, 1123–1135. 10.2174/1567205015666180801111033 30068274 PMC6302348

[bph16078-bib-0119] Marcello, E. , di Luca, M. , & Gardoni, F. (2018). Synapse‐to‐nucleus communication: From developmental disorders to Alzheimer's disease. Current Opinion in Neurobiology, 48, 160–166. 10.1016/j.conb.2017.12.017 29316492

[bph16078-bib-0120] Marcello, E. , Epis, R. , Saraceno, C. , Gardoni, F. , Borroni, B. , Cattabeni, F. , Padovani, A. , & di Luca, M. (2012). SAP97‐mediated local trafficking is altered in Alzheimer disease patients' hippocampus. Neurobiology of Aging, 33, 422.e1–422.e10. 10.1016/j.neurobiolaging.2010.09.015 20980075

[bph16078-bib-0121] Marcello, E. , Gardoni, F. , Mauceri, D. , Romorini, S. , Jeromin, A. , Epis, R. , Borroni, B. , Cattabeni, F. , Sala, C. , Padovani, A. , & di Luca, M. (2007). Synapse‐associated protein‐97 mediates α‐secretase ADAM10 trafficking and promotes its activity. The Journal of Neuroscience, 27, 1682–1691. 10.1523/JNEUROSCI.3439-06.2007 17301176 PMC6673742

[bph16078-bib-0122] Marcello, E. , Saraceno, C. , Musardo, S. , Vara, H. , de la Fuente, A. G. , Pelucchi, S. , di Marino, D. , Borroni, B. , Tramontano, A. , Pérez‐Otaño, I. , Padovani, A. , Giustetto, M. , Gardoni, F. , & di Luca, M. (2013). Endocytosis of synaptic ADAM10 in neuronal plasticity and Alzheimer's disease. The Journal of Clinical Investigation, 123, 2523–2538. 10.1172/JCI65401 23676497 PMC3668814

[bph16078-bib-0123] Marsh, S. E. , Abud, E. M. , Lakatos, A. , Karimzadeh, A. , Yeung, S. T. , Davtyan, H. , Fote, G. M. , Lau, L. , Weinger, J. G. , Lane, T. E. , Inlay, M. A. , Poon, W. W. , & Blurton‐Jones, M. (2016). The adaptive immune system restrains Alzheimer's disease pathogenesis by modulating microglial function. Proceedings of the National Academy of Sciences of the United States of America, 113, E1316–E1325. 10.1073/pnas.1525466113 26884167 PMC4780638

[bph16078-bib-0124] Martínez‐Iglesias, O. , Carrera, I. , Carril, J. C. , Fernández‐Novoa, L. , Cacabelos, N. , & Cacabelos, R. (2020). DNA methylation in neurodegenerative and cerebrovascular disorders. International Journal of Molecular Sciences, 21, 2220. 10.3390/ijms21062220 32210102 PMC7139499

[bph16078-bib-0125] Mastroeni, D. , Grover, A. , Delvaux, E. , Whiteside, C. , Coleman, P. D. , & Rogers, J. (2010). Epigenetic changes in Alzheimer's disease: Decrements in DNA methylation. Neurobiology of Aging, 31, 2025–2037. 10.1016/j.neurobiolaging.2008.12.005 19117641 PMC2962691

[bph16078-bib-0126] Mathys, H. , Davila‐Velderrain, J. , Peng, Z. , Gao, F. , Mohammadi, S. , Young, J. Z. , Menon, M. , He, L. , Abdurrob, F. , Jiang, X. , Martorell, A. J. , Ransohoff, R. M. , Hafler, B. P. , Bennett, D. A. , Kellis, M. , & Tsai, L. H. (2019). Single‐cell transcriptomic analysis of Alzheimer's disease. Nature, 570, 332–337. 10.1038/s41586-019-1195-2 31042697 PMC6865822

[bph16078-bib-0127] Mauceri, D. , Buchthal, B. , Hemstedt, T. J. , Weiss, U. , Klein, C. D. , & Bading, H. (2020). Nasally delivered VEGFD mimetics mitigate stroke‐induced dendrite loss and brain damage. Proceedings of the National Academy of Sciences, 117, 8616–8623. 10.1073/pnas.2001563117 PMC7165430 32229571

[bph16078-bib-0128] Mayne, K. , White, J. A. , McMurran, C. E. , Rivera, F. J. , & de la Fuente, A. G. (2020). Aging and neurodegenerative disease: Is the adaptive immune system a friend or foe? Frontiers in Aging Neuroscience, 12, 572090. 10.3389/fnagi.2020.572090 33173502 PMC7538701

[bph16078-bib-0129] Miller, R. G. , Mitchell, J. D. , Lyon, M. , & Moore, D. H. (2002). Riluzole for amyotrophic lateral sclerosis (ALS)/motor neuron disease (MND). Cochrane Database of Systematic Reviews, 3, CD001447. 10.1002/14651858.CD001447 12076411

[bph16078-bib-0130] Mora, J. S. , Genge, A. , Chio, A. , Estol, C. J. , Chaverri, D. , Hernández, M. , Marín, S. , Mascias, J. , Rodriguez, G. E. , Povedano, M. , Paipa, A. , Dominguez, R. , Gamez, J. , Salvado, M. , Lunetta, C. , Ballario, C. , Riva, N. , Mandrioli, J. , Moussy, A. , … the AB10015 STUDY GROUP . (2020). Masitinib as an add‐on therapy to riluzole in patients with amyotrophic lateral sclerosis: A randomized clinical trial. Amyotroph Lateral Scler Frontotemporal Degener, 21, 5–14. 10.1080/21678421.2019.1632346 31280619

[bph16078-bib-0131] Mullard, A. (2021). Targeted protein degraders crowd into the clinic. Nature Reviews. Drug Discovery, 20, 247–250. 10.1038/d41573-021-00052-4 33737725

[bph16078-bib-0132] Musardo, S. , Therin, S. , Pelucchi, S. , D'Andrea, L. , Stringhi, R. , Ribeiro, A. , Manca, A. , Balducci, C. , Pagano, J. , Sala, C. , Verpelli, C. , Grieco, V. , Edefonti, V. , Forloni, G. , Gardoni, F. , Meli, G. , di Marino, D. , di Luca, M. , & Marcello, E. (2022). The development of ADAM10 endocytosis inhibitors for the treatment of Alzheimer's disease. Molecular Therapy, 30, 2474–2490. 10.1016/j.ymthe.2022.03.024 35390543 PMC9263258

[bph16078-bib-0133] Neumann, B. , Baror, R. , Zhao, C. , Segel, M. , Dietmann, S. , Rawji, K. S. , Foerster, S. , McClain, C. R. , Chalut, K. , van Wijngaarden, P. , & Franklin, R. J. M. (2019). Metformin restores CNS remyelination capacity by rejuvenating aged stem cells. Cell Stem Cell, 25, 473–485.e8. 10.1016/j.stem.2019.08.015 31585093 PMC6863391

[bph16078-bib-0134] Niccoli, T. , & Partridge, L. (2012). Ageing as a risk factor for disease. Current Biology, 22, R741–R752. 10.1016/j.cub.2012.07.024 22975005

[bph16078-bib-0135] Nithianantharajah, J. , & Hannan, A. J. (2013). Dysregulation of synaptic proteins, dendritic spine abnormalities and pathological plasticity of synapses as experience‐dependent mediators of cognitive and psychiatric symptoms in Huntington's disease. Neuroscience, 251, 66–74. 10.1016/j.neuroscience.2012.05.043 22633949

[bph16078-bib-0136] Ogrodnik, M. , Evans, S. A. , Fielder, E. , Victorelli, S. , Kruger, P. , Salmonowicz, H. , Weigand, B. M. , Patel, A. D. , Pirtskhalava, T. , Inman, C. L. , & Johnson, K. O. (2021). Whole‐body senescent cell clearance alleviates age‐related brain inflammation and cognitive impairment in mice. Aging Cell, 20, e13296. 33470505 10.1111/acel.13296 PMC7884042

[bph16078-bib-0137] Olah, M. , Menon, V. , Habib, N. , Taga, M. F. , Ma, Y. , Yung, C. J. , Cimpean, M. , Khairallah, A. , Coronas‐Samano, G. , Sankowski, R. , Grün, D. , Kroshilina, A. A. , Dionne, D. , Sarkis, R. A. , Cosgrove, G. R. , Helgager, J. , Golden, J. A. , Pennell, P. B. , Prinz, M. , … de Jager, P. L. (2020). Single cell RNA sequencing of human microglia uncovers a subset associated with Alzheimer's disease. Nature Communications, 11, 6129. 10.1038/s41467-020-19737-2 PMC7704703 33257666

[bph16078-bib-0138] Onishi, T. , Maeda, R. , Terada, M. , Sato, S. , Fujii, T. , Ito, M. , Hashikami, K. , Kawamoto, T. , & Tanaka, M. (2021). A novel orally active HDAC6 inhibitor T‐518 shows a therapeutic potential for Alzheimer's disease and tauopathy in mice. Scientific Reports, 11, 15423. 10.1038/s41598-021-94923-w 34326423 PMC8322070

[bph16078-bib-0139] Orenstein, S. J. , Kuo, S.‐H. , Tasset, I. , Arias, E. , Koga, H. , Fernandez‐Carasa, I. , Cortes, E. , Honig, L. S. , Dauer, W. , Consiglio, A. , Raya, A. , Sulzer, D. , & Cuervo, A. M. (2013). Interplay of LRRK2 with chaperone‐mediated autophagy. Nature Neuroscience, 16, 394–406. 10.1038/nn.3350 23455607 PMC3609872

[bph16078-bib-0140] Palop, J. J. , Chin, J. , Roberson, E. D. , Wang, J. , Thwin, M. T. , Bien‐Ly, N. , Yoo, J. , Ho, K. O. , Yu, G. Q. , Kreitzer, A. , Finkbeiner, S. , Noebels, J. L. , & Mucke, L. (2007). Aberrant excitatory neuronal activity and compensatory remodeling of inhibitory hippocampal circuits in mouse models of Alzheimer's disease. Neuron, 55, 697–711. 10.1016/j.neuron.2007.07.025 17785178 PMC8055171

[bph16078-bib-0141] Papp, D. , Kovács, T. , Billes, V. , Varga, M. , Tarnóci, A. , Hackler, L. , Puskás, L. G. , Liliom, H. , Tárnok, K. , Schlett, K. , Borsy, A. , Pádár, Z. , Kovács, A. L. , Hegedűs, K. , Juhász, G. , Komlós, M. , Erdős, A. , Gulyás, B. , & Vellai, T. (2016). AUTEN‐67, an autophagy‐enhancing drug candidate with potent antiaging and neuroprotective effects. Autophagy, 12, 273–286. 10.1080/15548627.2015.1082023 26312549 PMC4835959

[bph16078-bib-0142] Pasciuto, E. , Burton, O. T. , Roca, C. P. , Lagou, V. , Rajan, W. D. , Theys, T. , Mancuso, R. , Tito, R. Y. , Kouser, L. , Callaerts‐Vegh, Z. , de la Fuente, A. G. , Prezzemolo, T. , Mascali, L. G. , Brajic, A. , Whyte, C. E. , Yshii, L. , Martinez‐Muriana, A. , Naughton, M. , Young, A. , … Liston, A. (2020). Microglia require CD4 T cells to complete the fetal‐to‐adult transition. Cell, 182, 625–640.e24. 10.1016/j.cell.2020.06.026 32702313 PMC7427333

[bph16078-bib-0143] Pickford, F. , Masliah, E. , Britschgi, M. , Lucin, K. , Narasimhan, R. , Jaeger, P. A. , Small, S. , Spencer, B. , Rockenstein, E. , Levine, B. , & Wyss‐Coray, T. (2008). The autophagy‐related protein beclin 1 shows reduced expression in early Alzheimer disease and regulates amyloid β accumulation in mice. The Journal of Clinical Investigation, 118, 2190–2199. 10.1172/JCI33585 18497889 PMC2391284

[bph16078-bib-0144] Pietronigro, E. , Zenaro, E. , Bianca, V. D. , Dusi, S. , Terrabuio, E. , Iannoto, G. , Slanzi, A. , Ghasemi, S. , Nagarajan, R. , Piacentino, G. , Tosadori, G. , Rossi, B. , & Constantin, G. (2019). Blockade of α4 integrins reduces leukocyte–endothelial interactions in cerebral vessels and improves memory in a mouse model of Alzheimer's disease. Scientific Reports, 9, 12055. 10.1038/s41598-019-48538-x 31427644 PMC6700124

[bph16078-bib-0145] Piette, F. , Belmin, J. , Vincent, H. , Schmidt, N. , Pariel, S. , Verny, M. , Marquis, C. , Mely, J. , Hugonot‐Diener, L. , Kinet, J. P. , Dubreuil, P. , Moussy, A. , & Hermine, O. (2011). Masitinib as an adjunct therapy for mild‐to‐moderate Alzheimer's disease: A randomised, placebo‐controlled phase 2 trial. Alzheimer's Research & Therapy, 3, 16. 10.1186/alzrt75 PMC3226277 21504563

[bph16078-bib-0146] Piscopo, P. , Crestini, A. , Carbone, E. , Rivabene, R. , Ancidoni, A. , Lo Giudice, M. , Corbo, M. , Vanacore, N. , & Lacorte, E. (2022). A systematic review on drugs for synaptic plasticity in the treatment of dementia. Ageing Research Reviews, 81, 101726. 10.1016/j.arr.2022.101726 36031056

[bph16078-bib-0147] Poewe, W. , Seppi, K. , Tanner, C. M. , Halliday, G. M. , Brundin, P. , Volkmann, J. , Schrag, A. E. , & Lang, A. E. (2017). Parkinson disease. Nature Reviews. Disease Primers, 3, 17013. 10.1038/nrdp.2017.13 28332488

[bph16078-bib-0148] Potenza, R. L. , de Simone, R. , Armida, M. , Mazziotti, V. , Pèzzola, A. , Popoli, P. , & Minghetti, L. (2016). Fingolimod: A disease‐modifier drug in a mouse model of amyotrophic lateral sclerosis. Neurotherapeutics, 13, 918–927. 10.1007/s13311-016-0462-2 27456702 PMC5081121

[bph16078-bib-0149] Prasad, H. , & Rao, R. (2018). Amyloid clearance defect in ApoE4 astrocytes is reversed by epigenetic correction of endosomal pH. Proceedings of the National Academy of Sciences, 115(28), E6640–E6649. 10.1073/pnas.1801612115 PMC6048470 29946028

[bph16078-bib-0150] Prince, H. M. , Bishton, M. J. , & Harrison, S. J. (2009). Clinical studies of histone deacetylase inhibitors. Clinical Cancer Research, 15, 3958–3969. 10.1158/1078-0432.CCR-08-2785 19509172

[bph16078-bib-0151] Pugazhenthi, S. , Wang, M. , Pham, S. , Sze, C.‐I. , & Eckman, C. B. (2011). Downregulation of CREB expression in Alzheimer's brain and in Aβ‐treated rat hippocampal neurons. Molecular Neurodegeneration, 6, 60. 10.1186/1750-1326-6-60 21854604 PMC3174124

[bph16078-bib-0152] Rawji, K. S. , Mishra, M. K. , Michaels, N. J. , Rivest, S. , Stys, P. K. , & Yong, V. W. (2016). Immunosenescence of microglia and macrophages: Impact on the ageing central nervous system. Brain, 139, 653–661. 10.1093/brain/awv395 26912633 PMC5839598

[bph16078-bib-0153] Rawji, K. S. , Neumann, B. , & Franklin, R. J. M. (2023). Glial aging and its impact on central nervous system myelin regeneration. Annals of the new York Academy of Sciences, 1519, 34–45. 10.1111/nyas.14933 36398864

[bph16078-bib-0154] Reddy, P. H. , Manczak, M. , & Yin, X. (2017). Mitochondria‐division inhibitor 1 protects against amyloid‐β induced mitochondrial fragmentation and synaptic damage in Alzheimer's disease. Journal of Alzheimer's Disease, 58, 147–162. 10.3233/JAD-170051 PMC5444307 28409745

[bph16078-bib-0155] Ricobaraza, A. , Cuadrado‐Tejedor, M. , Marco, S. , Pérez‐Otaño, I. , & García‐Osta, A. (2012). Phenylbutyrate rescues dendritic spine loss associated with memory deficits in a mouse model of Alzheimer disease. Hippocampus, 22, 1040–1050. 10.1002/hipo.20883 21069780

[bph16078-bib-0156] Rivera, A. D. , Pieropan, F. , Chacon‐De‐La‐Rocha, I. , Lecca, D. , Abbracchio, M. P. , Azim, K. , & Butt, A. M. (2021). Functional genomic analyses highlight a shift in *Gpr17*‐regulated cellular processes in oligodendrocyte progenitor cells and underlying myelin dysregulation in the aged mouse cerebrum. Aging Cell, 20, e13335. 33675110 10.1111/acel.13335 PMC8045941

[bph16078-bib-0157] Rizzuto, R. , de Stefani, D. , Raffaello, A. , & Mammucari, C. (2012). Mitochondria as sensors and regulators of calcium signalling. Nature Reviews. Molecular Cell Biology, 13, 566–578. 10.1038/nrm3412 22850819

[bph16078-bib-0158] Rockwood, K. , Hogan, D. B. , & MacKnight, C. (2000). Conceptualisation and measurement of frailty in elderly people. Drugs & Aging, 17, 295–302. 10.2165/00002512-200017040-00005 11087007

[bph16078-bib-0159] Rogers, J. T. , Morganti, J. M. , Bachstetter, A. D. , Hudson, C. E. , Peters, M. M. , Grimmig, B. A. , Weeber, E. J. , Bickford, P. C. , & Gemma, C. (2011). CX3CR1 deficiency leads to impairment of hippocampal cognitive function and synaptic plasticity. Journal of Neuroscience, 31, 16241–16250. 10.1523/JNEUROSCI.3667-11.2011 22072675 PMC3236509

[bph16078-bib-0160] Romero‐Miguel, D. , Lamanna‐Rama, N. , Casquero‐Veiga, M. , Gómez‐Rangel, V. , Desco, M. , & Soto‐Montenegro, M. L. (2021). Minocycline in neurodegenerative and psychiatric diseases: An update. European Journal of Neurology, 28, 1056–1081. 10.1111/ene.14642 33180965

[bph16078-bib-0161] Sá, J. v. , Kleiderman, S. , Brito, C. , Sonnewald, U. , Leist, M. , Teixeira, A. P. , & Alves, P. M. (2017). Quantification of metabolic rearrangements during neural stem cells differentiation into astrocytes by metabolic flux analysis. Neurochemical Research, 42, 244–253. 10.1007/s11064-016-1907-z 27068034

[bph16078-bib-0162] Safaiyan, S. , Kannaiyan, N. , Snaidero, N. , Brioschi, S. , Biber, K. , Yona, S. , Edinger, A. L. , Jung, S. , Rossner, M. J. , & Simons, M. (2016). Age‐related myelin degradation burdens the clearance function of microglia during aging. Nature Neuroscience, 19, 995–998. 10.1038/nn.4325 27294511 PMC7116794

[bph16078-bib-0163] Salvatore, F. (2020). The shift of the paradigm between ageing and diseases. Clinical Chemistry and Laboratory Medicine (CCLM), 58, 1635–1644. 10.1515/cclm-2020-0125 32286241

[bph16078-bib-0164] Sanchez, P. E. , Zhu, L. , Verret, L. , Vossel, K. A. , Orr, A. G. , Cirrito, J. R. , Devidze, N. , Ho, K. , Yu, G. Q. , Palop, J. J. , & Mucke, L. (2012). Levetiracetam suppresses neuronal network dysfunction and reverses synaptic and cognitive deficits in an Alzheimer's disease model. Proceedings of the National Academy of Sciences of the United States of America, 109, E2895–E2903. 10.1073/pnas.1121081109 22869752 PMC3479491

[bph16078-bib-0165] Sanchez‐Mut, J. V. , & Gräff, J. (2015). Epigenetic alterations in Alzheimer's disease. Frontiers in Behavioral Neuroscience, 9, 347. 26734709 10.3389/fnbeh.2015.00347 PMC4681781

[bph16078-bib-0166] Saraceno, C. , Marcello, E. , di Marino, D. , Borroni, B. , Claeysen, S. , Perroy, J. , Padovani, A. , Tramontano, A. , Gardoni, F. , & di Luca, M. (2014). SAP97‐mediated ADAM10 trafficking from Golgi outposts depends on PKC phosphorylation. Cell Death & Disease, 5, e1547. 10.1038/cddis.2014.492 25429624 PMC4260750

[bph16078-bib-0167] Sasaki, S. , & Iwata, M. (2007). Mitochondrial alterations in the spinal cord of patients with sporadic amyotrophic lateral sclerosis. Journal of Neuropathology and Experimental Neurology, 66, 10–16. 10.1097/nen.0b013e31802c396b 17204932

[bph16078-bib-0168] Sastre, M. , & Gentleman, S. M. (2010). NSAIDs: How they work and their prospects as therapeutics in Alzheimer's disease. Frontiers in Aging Neuroscience, 2, 20. 20589102 10.3389/fnagi.2010.00020 PMC2893374

[bph16078-bib-0169] Schafer, D. P. , Lehrman, E. K. , & Stevens, B. (2013). The “quad‐partite” synapse: Microglia‐synapse interactions in the developing and mature CNS. Glia, 61, 24–36. 10.1002/glia.22389 22829357 PMC4082974

[bph16078-bib-0170] Schlüter, A. , Aksan, B. , Diem, R. , Fairless, R. , & Mauceri, D. (2020). VEGFD protects retinal ganglion cells and, consequently, capillaries against excitotoxic injury. Mol Ther Methods Clin Dev, 17, 281–299. 10.1016/j.omtm.2019.12.009 32055648 PMC7005343

[bph16078-bib-0171] Selvarani, R. , Mohammed, S. , & Richardson, A. (2021). Effect of rapamycin on aging and age‐related diseases—Past and future. Geroscience, 43, 1135–1158. 10.1007/s11357-020-00274-1 33037985 PMC8190242

[bph16078-bib-0172] Sheean, R. K. , McKay, F. C. , Cretney, E. , Bye, C. R. , Perera, N. D. , Tomas, D. , Weston, R. A. , Scheller, K. J. , Djouma, E. , Menon, P. , Schibeci, S. D. , Marmash, N. , Yerbury, J. J. , Nutt, S. L. , Booth, D. R. , Stewart, G. J. , Kiernan, M. C. , Vucic, S. , & Turner, B. J. (2018). Association of regulatory T‐cell expansion with progression of amyotrophic lateral sclerosis: A study of humans and a transgenic mouse model. JAMA Neurology, 75, 681–689. 10.1001/jamaneurol.2018.0035 29507931 PMC5885208

[bph16078-bib-0173] Shinn, L. J. , & Lagalwar, S. (2021). Treating neurodegenerative disease with antioxidants: Efficacy of the bioactive phenol resveratrol and mitochondrial‐targeted MitoQ and SkQ. Antioxidants, 10, 573. 10.3390/antiox10040573 33917835 PMC8068221

[bph16078-bib-0174] Siciliano, G. , Carlesi, C. , Pasquali, L. , Piazza, S. , Pietracupa, S. , Fornai, F. , Ruggieri, S. , & Murri, L. (2010). Clinical trials for neuroprotection in ALS. CNS & Neurological Disorders Drug Targets, 9, 305–313. 10.2174/187152710791292648 20406180

[bph16078-bib-0175] Spilman, P. , Podlutskaya, N. , Hart, M. J. , Debnath, J. , Gorostiza, O. , Bredesen, D. , Richardson, A. , Strong, R. , & Galvan, V. (2010). Inhibition of mTOR by rapamycin abolishes cognitive deficits and reduces amyloid‐β levels in a mouse model of Alzheimer's disease. PLoS ONE, 5, e9979. 10.1371/journal.pone.0009979 20376313 PMC2848616

[bph16078-bib-0176] Spruston, N. , Stuart, G. , & Häusser, M. (2016). Principles of dendritic integration. In Dendrites (pp. 351–398). Oxford University Press.

[bph16078-bib-0177] Su, Q. , Li, T. , He, P.‐F. , Lu, X.‐C. , Yu, Q. , Gao, Q.‐C. , Wang, Z. J. , Wu, M. N. , Yang, D. , & Qi, J. S. (2021). Trichostatin A ameliorates Alzheimer's disease‐related pathology and cognitive deficits by increasing albumin expression and Aβ clearance in APP/PS1 mice. Alzheimer's Research & Therapy, 13, 7. 10.1186/s13195-020-00746-8 PMC7784383 33397436

[bph16078-bib-0178] Südhof, T. C. , & Malenka, R. C. (2008). Understanding synapses: Past, present, and future. Neuron, 60, 469–476. 10.1016/j.neuron.2008.10.011 18995821 PMC3243741

[bph16078-bib-0179] Sun, E. , Motolani, A. , Campos, L. , & Lu, T. (2022). The pivotal role of NF‐kB in the pathogenesis and therapeutics of Alzheimer's disease. International Journal of Molecular Sciences, 23, 8972. 10.3390/ijms23168972 36012242 PMC9408758

[bph16078-bib-0180] Talbott, E. O. , Malek, A. M. , & Lacomis, D. (2016). Chapter 13—The epidemiology of amyotrophic lateral sclerosis. In Handbook of clinical neurology (pp. 225–238). Elsevier. 10.1016/B978-0-12-802973-2.00013-6 27637961

[bph16078-bib-0181] Taylor, J. P. , Hardy, J. , & Fischbeck, K. H. (2002). Toxic proteins in neurodegenerative disease. Science, 296, 1991–1995. 12065827 10.1126/science.1067122

[bph16078-bib-0182] Thellung, S. , Corsaro, A. , Nizzari, M. , Barbieri, F. , & Florio, T. (2019). Autophagy activator drugs: A new opportunity in neuroprotection from misfolded protein toxicity. International Journal of Molecular Sciences, 20, 901. 10.3390/ijms20040901 30791416 PMC6412775

[bph16078-bib-0183] Thompson, K. R. , Otis, K. O. , Chen, D. Y. , Zhao, Y. , O'Dell, T. J. , & Martin, K. C. (2004). Synapse to nucleus signaling during long‐term synaptic plasticity: A role for the classical active nuclear import pathway. Neuron, 44, 997–1009. 10.1016/j.neuron.2004.11.025 15603742

[bph16078-bib-0184] Thompson, R. E. , Tuchman, A. J. , & Alkon, D. L. (2022). Bryostatin placebo‐controlled trials indicate cognitive restoration above baseline for advanced Alzheimer's disease in the absence of memantine^1^ . Journal of Alzheimer's Disease, 86, 1221–1229. 10.3233/JAD-215545 PMC9108553 35124654

[bph16078-bib-0185] Tintore, M. , Vidal‐Jordana, A. , & Sastre‐Garriga, J. (2019). Treatment of multiple sclerosis—Success from bench to bedside. Nature Reviews. Neurology, 15, 53–58. 10.1038/s41582-018-0082-z 30315270

[bph16078-bib-0186] Togo, T. , Akiyama, H. , Iseki, E. , Kondo, H. , Ikeda, K. , Kato, M. , Oda, T. , Tsuchiya, K. , & Kosaka, K. (2002). Occurrence of T cells in the brain of Alzheimer's disease and other neurological diseases. Journal of Neuroimmunology, 124, 83–92. 10.1016/S0165-5728(01)00496-9 11958825

[bph16078-bib-0216] Tracy, T. E. , Madero‐Pérez, J. , Swaney, D. L. , Chang, T. S. , Moritz, M. , Konrad, C. , Ward, M. E. , Stevenson, E. , Hüttenhain, R. , Kauwe, G. , Mercedes, M. , Sweetland‐Martin, L. , Chen, X. , Mok, S. A. , Wong, M. Y. , Telpoukhovskaia, M. , Min, S. W. , Wang, C. , Sohn, P. D. , … Gan, L. (2022). Tau interactome maps synaptic and mitochondrial processes associated with neurodegeneration. Cell, 185(4), 712‐728.e14. 10.1016/j.cell.2021.12.041 35063084 PMC8857049

[bph16078-bib-0187] Traxler, L. , Herdy, J. R. , Stefanoni, D. , Eichhorner, S. , Pelucchi, S. , Szücs, A. , Santagostino, A. , Kim, Y. , Agarwal, R. K. , Schlachetzki, J. C. M. , Glass, C. K. , Lagerwall, J. , Galasko, D. , Gage, F. H. , D'Alessandro, A. , & Mertens, J. (2022). Warburg‐like metabolic transformation underlies neuronal degeneration in sporadic Alzheimer's disease. Cell Metabolism, 34, 1248–1263.e6. 10.1016/j.cmet.2022.07.014 35987203 PMC9458870

[bph16078-bib-0188] Traxler, L. , Lagerwall, J. , Eichhorner, S. , Stefanoni, D. , D'Alessandro, A. , & Mertens, J. (2021). Metabolism navigates neural cell fate in development, aging and neurodegeneration. Disease Models & Mechanisms, 14(8), dmm048993. 10.1242/dmm.048993 34345916 PMC8353098

[bph16078-bib-0189] Verkhratsky, A. , & Nedergaard, M. (2018). Physiology of astroglia. Physiological Reviews, 98, 239–389. 10.1152/physrev.00042.2016 29351512 PMC6050349

[bph16078-bib-0190] Verma, M. , Lizama, B. N. , & Chu, C. T. (2022). Excitotoxicity, calcium and mitochondria: A triad in synaptic neurodegeneration. Transl Neurodegener, 11, 3. 10.1186/s40035-021-00278-7 35078537 PMC8788129

[bph16078-bib-0191] Vezzoli, E. , Caron, I. , Talpo, F. , Besusso, D. , Conforti, P. , Battaglia, E. , Sogne, E. , Falqui, A. , Petricca, L. , Verani, M. , Martufi, P. , Caricasole, A. , Bresciani, A. , Cecchetti, O. , Rivetti di Val Cervo, P. , Sancini, G. , Riess, O. , Nguyen, H. , Seipold, L. , … Zuccato, C. (2019). Inhibiting pathologically active ADAM10 rescues synaptic and cognitive decline in Huntington's disease. The Journal of Clinical Investigation, 129, 2390–2403. 10.1172/JCI120616 31063986 PMC6546448

[bph16078-bib-0192] Walsh, D. M. , Klyubin, I. , Fadeeva, J. v. , Cullen, W. K. , Anwyl, R. , Wolfe, M. S. , Rowan, M. J. , & Selkoe, D. J. (2002). Naturally secreted oligomers of amyloid β protein potently inhibit hippocampal long‐term potentiation in vivo. Nature, 416, 535–539. 10.1038/416535a 11932745

[bph16078-bib-0193] Wang, D. , & Hiesinger, P. R. (2012). Autophagy, neuron‐specific degradation and neurodegeneration. Autophagy, 8, 711–713. 10.4161/auto.19660 22498474 PMC3405846

[bph16078-bib-0194] Wang, F. , Ren, S.‐Y. , Chen, J.‐F. , Liu, K. , Li, R.‐X. , Li, Z.‐F. , Hu, B. , Niu, J. Q. , Xiao, L. , Chan, J. R. , & Mei, F. (2020). Myelin degeneration and diminished myelin renewal contribute to age‐related deficits in memory. Nature Neuroscience, 23, 481–486. 10.1038/s41593-020-0588-8 32042174 PMC7306053

[bph16078-bib-0195] Wang, X. , Becker, K. , Levine, N. , Zhang, M. , Lieberman, A. P. , Moore, D. J. , & Ma, J. (2019). Pathogenic alpha‐synuclein aggregates preferentially bind to mitochondria and affect cellular respiration. Acta Neuropathologica Communications, 7, 41. 10.1186/s40478-019-0696-4 30871620 PMC6419482

[bph16078-bib-0196] Wang, X. , Su, B. , Lee, H. G. , Li, X. , Perry, G. , Smith, M. A. , & Zhu, X. (2009). Impaired balance of mitochondrial fission and fusion in Alzheimer's disease. Journal of Neuroscience, 29, 9090–9103. 10.1523/JNEUROSCI.1357-09.2009 19605646 PMC2735241

[bph16078-bib-0197] Wang, X. , Su, B. , Siedlak, S. L. , Moreira, P. I. , Fujioka, H. , Wang, Y. , Casadesus, G. , & Zhu, X. (2008). Amyloid‐β overproduction causes abnormal mitochondrial dynamics via differential modulation of mitochondrial fission/fusion proteins. Proceedings of the National Academy of Sciences, 105, 19318–19323. 10.1073/pnas.0804871105 PMC2614759 19050078

[bph16078-bib-0198] Winner, B. , Jappelli, R. , Maji, S. K. , Desplats, P. A. , Boyer, L. , Aigner, S. , Hetzer, C. , Loher, T. , Vilar, M. , Campioni, S. , Tzitzilonis, C. , Soragni, A. , Jessberger, S. , Mira, H. , Consiglio, A. , Pham, E. , Masliah, E. , Gage, F. H. , & Riek, R. (2011). In vivo demonstration that α‐synuclein oligomers are toxic. Proceedings of the National Academy of Sciences, 108, 4194–4199. 10.1073/pnas.1100976108 PMC3053976 21325059

[bph16078-bib-0199] Wu, D. C. , Jackson‐Lewis, V. , Vila, M. , Tieu, K. , Teismann, P. , Vadseth, C. , Choi, D. K. , Ischiropoulos, H. , & Przedborski, S. (2002). Blockade of microglial activation is neuroprotective in the 1‐methyl‐4‐phenyl‐1,2,3,6‐tetrahydropyridine mouse model of Parkinson disease. The Journal of Neuroscience, 22, 1763–1771. 10.1523/JNEUROSCI.22-05-01763.2002 11880505 PMC6758858

[bph16078-bib-0200] Xia, Q. , Wang, H. , Hao, Z. , Fu, C. , Hu, Q. , Gao, F. , Ren, H. , Chen, D. , Han, J. , Ying, Z. , & Wang, G. (2016). TDP‐43 loss of function increases TFEB activity and blocks autophagosome–lysosome fusion. The EMBO Journal, 35, 121–142. 10.15252/embj.201591998 26702100 PMC4718457

[bph16078-bib-0201] Xin, W. , & Chan, J. R. (2020). Myelin plasticity: Sculpting circuits in learning and memory. Nature Reviews. Neuroscience, 21, 682–694. 10.1038/s41583-020-00379-8 33046886 PMC8018611

[bph16078-bib-0202] Xu, X. , He, X. , Zhang, Z. , Chen, Y. , Li, J. , Ma, S. , Huang, Q. , & Li, M. (2022). CREB inactivation by HDAC1/PP1γ contributes to dopaminergic neurodegeneration in Parkinson's disease. The Journal of Neuroscience, 42, 4594–4604. 10.1523/JNEUROSCI.1419-21.2022 35501151 PMC9172078

[bph16078-bib-0203] Yadav, E. , Yadav, P. , Khan, M. M. U. , Singh, H. , & Verma, A. (2022). Resveratrol: A potential therapeutic natural polyphenol for neurodegenerative diseases associated with mitochondrial dysfunction. Frontiers in Pharmacology, 13, 922232. 10.3389/fphar.2022.922232 36188541 PMC9523540

[bph16078-bib-0204] Yamamoto, A. , & Simonsen, A. (2011). The elimination of accumulated and aggregated proteins: A role for aggrephagy in neurodegeneration. Neurobiology of Disease, 43, 17–28. 10.1016/j.nbd.2010.08.015 20732422 PMC2998573

[bph16078-bib-0205] Yang, Y. , Coleman, M. , Zhang, L. , Zheng, X. , & Yue, Z. (2013). Autophagy in axonal and dendritic degeneration. Trends in Neurosciences, 36, 418–428. 10.1016/j.tins.2013.04.001 23639383 PMC3787524

[bph16078-bib-0206] Youle, R. J. , & Narendra, D. P. (2011). Mechanisms of mitophagy. Nature Reviews. Molecular Cell Biology, 12, 9–14. 10.1038/nrm3028 21179058 PMC4780047

[bph16078-bib-0207] Yshii, L. , Pasciuto, E. , Bielefeld, P. , Mascali, L. , Lemaitre, P. , Marino, M. , Dooley, J. , Kouser, L. , Verschoren, S. , Lagou, V. , Kemps, H. , Gervois, P. , de Boer, A. , Burton, O. T. , Wahis, J. , Verhaert, J. , Tareen, S. H. K. , Roca, C. P. , Singh, K. , … Liston, A. (2022). Astrocyte‐targeted gene delivery of interleukin 2 specifically increases brain‐resident regulatory T cell numbers and protects against pathological neuroinflammation. Nature Immunology, 23, 878–891. 10.1038/s41590-022-01208-z 35618831 PMC9174055

[bph16078-bib-0208] Zambon, F. , Cherubini, M. , Fernandes, H. J. R. , Lang, C. , Ryan, B. J. , Volpato, V. , Bengoa‐Vergniory, N. , Vingill, S. , Attar, M. , Booth, H. D. E. , Haenseler, W. , Vowles, J. , Bowden, R. , Webber, C. , Cowley, S. A. , & Wade‐Martins, R. (2019). Cellular α‐synuclein pathology is associated with bioenergetic dysfunction in Parkinson's iPSC‐derived dopamine neurons. Human Molecular Genetics, 28, 2001–2013. 10.1093/hmg/ddz038 30753527 PMC6548224

[bph16078-bib-0209] Zhang, P. , Kishimoto, Y. , Grammatikakis, I. , Gottimukkala, K. , Cutler, R. G. , Zhang, S. , Abdelmohsen, K. , Bohr, V. A. , Misra Sen, J. , Gorospe, M. , & Mattson, M. P. (2019). Senolytic therapy alleviates Aβ‐associated oligodendrocyte progenitor cell senescence and cognitive deficits in an Alzheimer's disease model. Nature Neuroscience, 22, 719–728. 10.1038/s41593-019-0372-9 30936558 PMC6605052

[bph16078-bib-0210] Zhao, P. , Yang, X. , Yang, L. , Li, M. , Wood, K. , Liu, Q. , & Zhu, X. (2017). Neuroprotective effects of fingolimod in mouse models of Parkinson's disease. The FASEB Journal, 31, 172–179. 10.1096/fj.201600751r 27671228

[bph16078-bib-0211] Zhelev, Z. , Bakalova, R. , Aoki, I. , Lazarova, D. , & Saga, T. (2013). Imaging of superoxide generation in the dopaminergic area of the brain in Parkinson's disease, using Mito‐TEMPO. ACS Chemical Neuroscience, 4, 1439–1445. 10.1021/cn400159h 24024751 PMC3837371

[bph16078-bib-0212] Ziv, Y. , Ron, N. , Butovsky, O. , Landa, G. , Sudai, E. , Greenberg, N. , Cohen, H. , Kipnis, J. , & Schwartz, M. (2006). Immune cells contribute to the maintenance of neurogenesis and spatial learning abilities in adulthood. Nature Neuroscience, 9, 268–275. 10.1038/nn1629 16415867

[bph16078-bib-0213] Zoing, M. C. , Burke, D. , Pamphlett, R. , & Kiernan, M. C. (2006). Riluzole therapy for motor neurone disease: An early Australian experience (1996–2002). Journal of Clinical Neuroscience, 13, 78–83. 10.1016/j.jocn.2004.04.011 16410201

